# Unscented Kalman filter for airship model uncertainties and wind disturbance estimation

**DOI:** 10.1371/journal.pone.0257849

**Published:** 2021-11-05

**Authors:** Muhammad Wasim, Ahsan Ali, Mohammad Ahmad Choudhry, Faisal Saleem, Inam Ul Hasan Shaikh, Jamshed Iqbal

**Affiliations:** 1 Department of Electrical Engineering, University of Engineering and Technology, Taxila, Pakistan; 2 Department of Electrical Engineering, Riphah International University, Islamabad, Pakistan; 3 Department of Computer Science and Technology, Faculty of Science and Engineering, University of Hull, Hull, United Kingdom; Effat University, SAUDI ARABIA

## Abstract

An airship is lighter than an air vehicle with enormous potential in applications such as communication, aerial inspection, border surveillance, and precision agriculture. An airship model is made up of dynamic, aerodynamic, aerostatic, and propulsive forces. However, the computation of aerodynamic forces remained a challenge. In addition to aerodynamic model deficiencies, airship mass matrix suffers from parameter variations. Moreover, due to the lighter-than-air nature, it is also susceptible to wind disturbances. These modeling issues are the key challenges in developing an efficient autonomous flight controller for an airship. This article proposes a unified estimation method for airship states, model uncertainties, and wind disturbance estimation using Unscented Kalman Filter (UKF). The proposed method is based on a lumped model uncertainty vector that unifies model uncertainties and wind disturbances in a single vector. The airship model is extended by incorporating six auxiliary state variables into the lumped model uncertainty vector. The performance of the proposed methodology is evaluated using a nonlinear simulation model of a custom-developed UETT airship and is validated by conducting a kind of error analysis. For comparative studies, EKF estimator is also developed. The results show the performance superiority of the proposed estimator over EKF; however, the proposed estimator is a bit expensive on computational grounds. However, as per the requirements of the current application, the proposed estimator can be a preferred choice.

## 1. Introduction

An airship is a lighter-than-air, buoyancy-driven vehicle that gains its lift from low-density gas such as helium or hydrogen. An airship can be constructed using a rigid frame, or it may consist of a flexible envelope. To make it fly worthy, a comprehensive steering mechanism and a useful payload are attached to it. An airship has some unique and promising characteristics, making it a favorite among air vehicles, which led to its reemergence after 60 years of silence. Now, many companies and institutions worldwide are enthusiastically working on manned and unmanned airship-based projects [1]. An airship has certain advantages over conventional air vehicles, for example, environmentally friendly vehicle, simpler technology, ease of operation, low power consumption, and long-duration flights. These advantages make them suitable for many potential applications. For example, hovering capabilities make it suitable for a high-altitude communication platform. It has high payload carrying capabilities and can provide a long endurance platform for military surveillance, environmental and agricultural monitoring, advertisement, geographical surveys, and data collection for research [2–4].

These applications require various autonomous flight missions. For the successful execution of these missions, developing an efficient and reliable navigation and control system for an airship is inevitable. However, the complex dynamics of an airship and its vulnerability to wind disturbances pose a great challenge for designing an efficient control system. In literature, many nonlinear control approaches, such as Sliding Mode Control (SMC) [5, 6], Back-Stepping Controller (BSC) [7], Model Predictive Control [8, 9], and Nonlinear Model Predictive Controller (NMPC) [10], have been applied for the development of a control system for an airship and ground vehicles. Although they are robust control methods, they are usually designed for the worst-case scenario, sacrificing the nominal performance. They perform satisfactorily well if the model uncertainty prevails within specified bounds for which the controller is designed. An airship model suffers from model uncertainties that may degrade the performance of the nonlinear control methods if the uncertainty exceeds the assumed bounds. In the literature, researchers have suggested a robust control method. For example, in [11], the author has designed the scenario-based H-infinity controller for the ground vehicles to handle uncertainty. In hybrid control methods that couple the neural networks, adaptive and fuzzy methods with SMC or BSC are used to handle airship model uncertainties and increase the overall control performance [12–15].

Hence, it is important to provide reasonably accurate model information to the controller for adequate control performance. An airship model is made up of dynamic, aerodynamic, aerostatic, and propulsion forces. Apart from others, the computation of aerodynamic forces remains an issue for dynamic airship analysis [16]. In the literature, wind tunnel experiments, computational aerodynamic methods, and nonlinear estimation methods have been used for aerodynamic model computation. In wind tunnel experiments, an airship-scaled physical model is mounted at a fixed location in the tunnel with pressure measuring sensors at different locations on its surface. The wind is blown on it at different angles of attack and sideslip angles. From the sensor’s data, drag force, side force, lift force, rolling, pitching, and yaw moment coefficient are computed [17]. Wind tunnel methods were common in the early days of airship development when other methods were not available [18]. Many reports of the national advisory committee for aeronautics have covered the wind tunnel data for many British and American airships [19, 20]. Wind tunnel experiments were also used to develop an aerodynamic model for modern airships; for example, Gomes has developed the aerodynamics of the YEZ-2A airship by collecting 600 hours of wind tunnel data for different sideslip and angle of attacks [17]. Jones, from the University of Toronto Institute for Aerospace Studies, has performed wind tunnel experiments for TCOM-250 aerostat [21]. Wind tunnel data for Lotte airship developed in the University of Stuttgart, Germany, have also been reported in the literature [22]. However, these experimental setups are expensive and the collection of wind tunnel data for a large range of angle of attacks and sideslip angle is a laborious task. Consequently, researchers have proposed aerodynamic models that depend on airship geometrical parameters.

Initial theoretical work on the airship aerodynamic model can be found in the late 1920s. In this context, Munk has published a report that discussed airship aerodynamic characteristics using potential flow theory [23]. Munk’s work is improved by the incorporation of the crossflow drag [24]. Later, aerodynamic modeling equations are modified by incorporating airship hull and fin interaction [21]. Muller has formulated the 6-degree-of-freedom (DOF) nonlinear equations for calculating airship aerodynamic forces and torques, but these equations were deficient in damping terms due to roll, pitch, and yaw moments [25]. Muller’s work is improved by incorporating damping terms in the nonlinear equations [16]. These methods depend on the geometrical parameters of an airship and assume that the envelope maintains its shape. However, aerodynamic forces may vary due to unforeseen shape changes in the envelope.

Some researchers have suggested nonlinear filter-based estimation approaches to avoid the expensive wind tunnel experiments and address the limitations of computational aerodynamic calculation methods. They have estimated the aerodynamic coefficients or the complete aerodynamic model. In these estimation methods, Kalman filters have been applied to estimate aerodynamic model coefficients [26] or aerodynamic forces and torques [27, 28]. The aerodynamic model parameter estimation method estimates more than 50 parameters [26]. That makes the procedure computationally exhaustive because it introduces more than 50 augmented state variables, resulting in many states. This is directly related to the Kalman filter computational complexity. However, the method suggested by some researchers introduces only six augmented state variables to estimate the complete aerodynamic model [27, 28].

These estimation methods are a good cost-effective solution for approximating the airship aerodynamic model. However, they do not consider the model uncertainties due to variation in the airship mass matrix parameters and wind disturbances. Parameter variations of airship mass matrix and dynamic forces are inevitable. Airship mass matrix and dynamic forces consist of airship mass, added mass, inertia, added inertia, and Center of Gravity (CG) terms [29]. During airship flight, the air is charged in and out of the envelope, which causes changes in mass matrix parameters. Even a small amount of helium gas leakage also changes mass matrix parameters. Apart from model uncertainties, due to the lighter-than-air nature, an airship is also susceptible to wind disturbances. In the literature, adaptive neural networks and fuzzy methods are used for handling airship model uncertainties and wind disturbances [12–15]. Nevertheless, they are computationally intensive.

Building on the existing literature, in this paper, an estimation solution is proposed that introduces a lumped model uncertainties estimation approach based on the UKF estimator. The proposed work is a significant extension of our previous results reported in [27, 28]. The previous work only deals with the estimation of an airship aerodynamic model; however, the proposed work extends the results and incorporates model uncertainties and wind disturbances at the same time. The method estimates the airship states and the combined uncertainty vector incorporating mass matrix variations, aerodynamic model deficiencies, and wind forces. The proposed method avoids the expensive wind tunnel experimentation, deficiencies, and limitation of existing computational aerodynamic methods and the aerodynamic model estimation methods. The estimated uncertainty vector can be used to enhance the performance of a model-based controller. The proposed approach is validated by conducting extensive simulations and considering three cases where the lumped model uncertainty vector estimates the mass matrix variations, aerodynamic model deficiencies, and wind disturbances. For the comparative study, the same problem is solved using the EKF estimator.

## 2. Airship modeling

**Fig 1:**
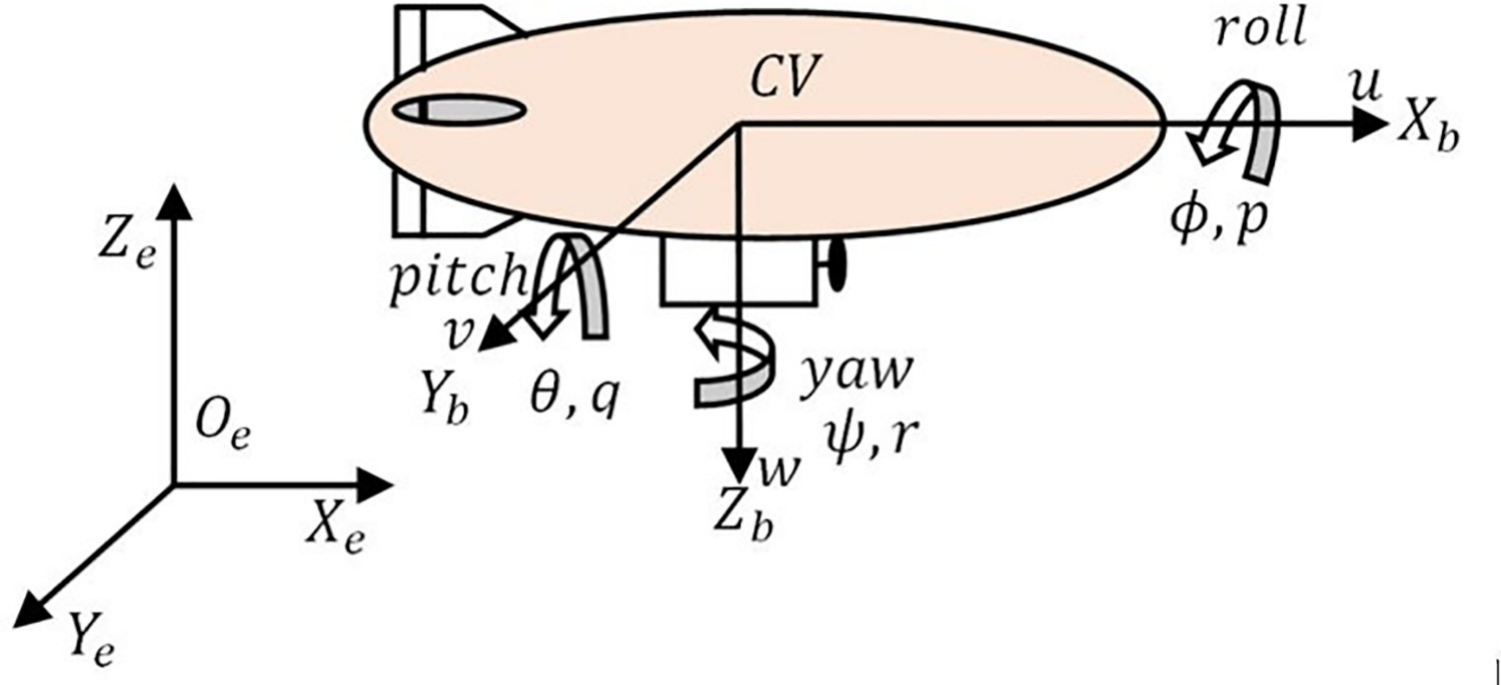
The coordinate system of an airship.

An airship is made up of an axis-symmetric, teardrop-shaped hull filled with helium gas, as shown in Fig 1. Its envelope accommodates an air-filled ballonet that controls the buoyancy. Two rudders and elevators are mounted on its tail in a plus configuration that creates the necessary aerodynamic force for maneuvering during an airship cruise flight. It is equipped with propellers mounted on both sides of the gondola. Gondola houses batteries, cameras, navigation, and control equipment required for autonomous airship operation.

As the airship is a buoyancy-driven air vehicle, its equations of motion are slightly different from the conventional aircraft. Because of its lighter-than-air nature, the main difference is the added mass and inertial terms. Underwater vehicles and airships are both buoyancy-driven vehicles. So, the mathematical model of an airship is derived from well-developed models of Remotely Operated underwater Vehicles (ROVs). Gomes [17] has used the small perturbation model of ROVs to study the YEZ-2A airship flight dynamics. He has adopted the 6-DOF nonlinear model of an airship, which is comprised of dynamics, buoyancy, gravity, aerodynamic, and propulsion forces. Because an airship is prone to wind disturbances, wind forces are also incorporated into the airship model [30].

Two reference frames are used for assessing airship navigation. As shown in Fig 1, an inertial reference frame located at any fixed point on the surface of the earth is denoted by *O*_*e*_*X*_*e*_*Y*_*e*_*Z*_*e*_, where *O*_*e*_*X*_*e*_-axis points toward the north, *O*_*e*_*Y*_*e*_-axis points toward the east, and *O*_*e*_*Z*_*e*_-axis points upward. A body-fixed reference frame is centered at the Center of Volume (CV) of the airship, denoted by *O*_*b*_*X*_*b*_*Y*_*b*_*Z*_*b*_, where *O*_*b*_*X*_*b*_-axis points toward its nose. It coincides with the symmetry axis of the envelope, *O*_*b*_*Z*_*b*_-axis points toward the earth center and *O*_*b*_*Y*_*b*_-axis as determined by the right-hand rule points to the right.

Generalized coordinates of an airship are represented by $\bar{\xi }=[P,\Phi ]\prime$, where P = [*x*,*y*,*z*]′ is the position with respect to the inertial frame and Φ = [*ϕ*,*θ*,*ψ*]′ are its attitudes. The generalized vector for velocities with respect to the body frame is represented by ${\bar{V}}_{b}=[\nu ,\Omega ]\prime$, where *v* = [*u*,*v*,*w*]′ is the vector of linear velocities and Ω = [*p*,*q*,*r*]′ are the angular velocities. Using these notations, the vector form of the 6-DOF equation of motion for an airship can be written as follows:

$$\bar{\dot{\xi }}=R(\Phi ){\bar{V}}_{b}$$ (1)

$${\bar{\dot{V}}}_{b}={\bar{M}}^{-1}({\bar{F}}_{D}+{\bar{F}}_{AS}+{\bar{F}}_{AD}+F_{W}+U),$$ (2)

where $\bar{\xi }=[x,y,z,\phi ,\theta ,\psi ]$ and ${\bar{V}}_{b}=[u,v,w,p,q,r]$. *R*(Φ) = diag(*R*_1_(Φ), *R*_2_(Φ)) is the rotation matrix that transforms the body axes velocities to the inertial frame; $\bar{M}$ is the airship mass matrix; ${\bar{F}}_{D}$ is the dynamic force vector that is made up of Coriolis and centripetal forces; ${\bar{F}}_{AS}$ is the aerostatic force vector that is made up of buoyancy and gravity forces; ${\bar{F}}_{AD}$ is the aerodynamic force vector; *U* is the generalized control input vector. The expressions for rotation matrix with the simplification of cos(.), sin(.) and tan(.) as c_(.)_, s_(.)_, t_(.)_, respectively, are given in the following equations:

$$R_{1}(\Phi )=\left[\begin{array}{ccc} c_{\psi }c_{\theta } & c_{\psi }s_{\theta }s_{\phi }-s_{\psi }c_{\phi } & c_{\psi }s_{\theta }c_{\phi }+s_{\psi }s_{\phi }\\ s_{\psi }c_{\theta } & s_{\theta }s_{\phi }s_{\psi }+c_{\psi }c_{\phi } & s_{\psi }c_{\phi }s_{\theta }-c_{\psi }s_{\phi }\\ -s_{\theta } & c_{\theta }s_{\psi } & c_{\theta }c_{\psi } \end{array}\right],$$ (3)

$$R_{2}(\Phi )=[\begin{array}{ccc} 1 & s_{\phi }t_{\theta } & c_{\phi }t_{\theta }\\ 0 & c_{\phi } & -s_{\phi }\\ 0 & s_{\phi }\sec\left(\theta \right) & c_{\phi }\sec\left(\theta \right) \end{array}].$$ (4)

The mass matrix is given as follows:

$$\bar{M}=\left[\begin{array}{cccccc} m_{1} & 0 & 0 & 0 & m_{5} & 0\\ 0 & m_{2} & 0 & m_{4} & 0 & m_{6}\\ 0 & 0 & m_{3} & 0 & m_{7} & 0\\ 0 & m_{8} & 0 & m_{9} & 0 & m_{10}\\ m_{11} & 0 & m_{12} & 0 & m_{13} & 0\\ 0 & m_{14} & 0 & m_{15} & 0 & m_{16} \end{array}\right].$$ (5)

The mass matrix terms are defined in S1 Appendix. They are made up of airship mass, added mass, inertia, added inertia, and its CG. The dynamic, aerodynamic, and aerostatic force vectors are given in the following equations:

$${\bar{F}}_{D}=[\begin{array}{c} -m_{z}wq+m_{y}rv+m[a_{x}(q^{2}+r^{2})-a_{z}rp]\\ -m_{x}ur+m_{z}pw+m[-a_{x}pq-a_{z}rq]\\ -m_{y}vp+m_{x}qu+m[-a_{x}rp+a_{z}(q^{2}+p^{2})]\\ -(J_{z}-J_{y})rq+J_{xz}pq+ma_{z}(ur-pw)\\ -(J_{x}-J_{z})pr+J_{xz}(r^{2}-p^{2})+\ldots \\ m[a_{x}(vp-qu)-a_{z}(wq-rv)]\\ -(J_{y}-J_{x})qp-J_{xz}qr+m[-a_{x}(ur-pw)] \end{array}],$$ (6)

F¯AD=f(Vt)[CX1cα2cβ2+CX2s2αsα2CY1cβ2s2β+CY2s2β+CY3sβs|β|Cz1cα2s2α+Cz2s2α+Cz3sαs|α|CL2sβs|β|CM1cα2s2α+CM2s2α+CM3sαs|α|CN1cβ2s2β+CN2s2β+CN3sβs|β|], (7)

$${\bar{F}}_{AS}=\left[\begin{array}{c} -(W-B_{f})s_{\theta }\\ (W-B_{f})c_{\theta }s_{\phi }\\ (W-B_{f})c_{\theta }c_{\phi }\\ a_{z}Wc_{\theta }s_{\phi }\\ -{(a}_{z}W-b_{z}B_{f})s_{\theta }-{(a}_{x}W-b_{x}B_{f})c_{\theta }c_{\phi }\\ a_{x}Wc_{\theta }s_{\phi } \end{array}\right],$$ (8)

where *m*, *m*_*x*_, *m*_*y*_, *m*_*z*_, *J*_*x*_, *J*_*y*_, *J*_*z*_, *J*_*xz*_ are the terms corresponding to the airship mass and inertia and are given in S1 Appendix. (*a*_*x*_, *a*_*z*_) are the coordinates of CG with respect to CV. *α* and *β* are the angle of attack and sideslip angles, respectively. *W* is the airship weight and *B*_*f*_ is the buoyancy force. The function f(Vt)=12ρVt2 and *V*_*t*_ is the velocity of airship. *C*_*ij*_(*i* = *X*,*Y*,*Z*,*L*,*M*,*N*;*j* = 1,2,3,4) are the aerodynamic coefficients.

It is considered that wind is acting on an airship in a horizontal inertial plane with constant velocity. It does not induce angular disturbance. The wind velocity in the body frame can be obtained by transforming wind speed from the inertial frame using the transformation matrix defined in (3). So, the wind velocity in the body frame can be expressed by the following equation:

$$V_{w}=R(\Phi )[\begin{array}{c} V_{Nw}\cos\left(\psi_{w}\right)\\ V_{EW}\sin\left(\psi_{w}\right)\\ 0 \end{array}],$$ (9)

where *V*_*Nw*_ and *V*_*EW*_ are the wind velocities in the inertial frame with an incidence angle of *ψ*_*w*_ with respect to the inertial frame. It has the same sign convention as the yaw angle defined earlier. The Dryden model power spectral density function is used to model the turbulent gust [31]. Gust velocities are generated by applying noise inputs having unitary power spectral density function to the filters given in the following equation:

$$H_{u}(s)=\sqrt{\frac{2V_{e}\sigma_{u}^{2}}{L_{u}\pi }}[\frac{1}{s+(\frac{V_{e}}{L_{u}})}],$$ (10)

$$H_{v}(s)=\sqrt{\frac{2V_{e}\sigma_{v}^{2}}{L_{v}\pi }}[\frac{s+(\frac{V_{e}}{{\sqrt{3}L}_{u}})}{{\left(s+(\frac{V_{e}}{L_{v}})\right)}^{2}}],$$ (11)

$$H_{w}(s)=\sqrt{\frac{2\sigma_{w}^{2}}{L_{w}V_{e}\pi }}\left[\frac{s+(\frac{V_{e}}{{\sqrt{3}L}_{w}})}{{\left(s+(\frac{V_{e}}{L_{w}})\right)}^{2}}\right],$$ (12)

where {*L*_*u*_, *L*_*v*_, *L*_*w*_} are the turbulence scale lengths that depend on aircraft height. {*σ*_*u*_, *σ*_*v*_, *σ*_*w*_} are the intensities of turbulence in each direction. ‘*s*’ is the Laplace operator and *V*_*e*_ is the equilibrium speed of aircraft. The filters output the translational velocities {*u*_*g*_, *v*_*g*_, *w*_*g*_} of the atmospheric gusts. If the linear turbulence gust velocity vector is represented by *V*_*g*_ = [*u*_*g*_ *v*_*g*_ *w*_*g*_]^*T*^, then the airflow vector can be given by *V*_*a*_ = *V*_*g*_+*V*_*w*_. In this work, it is assumed that the turbulence gust does not produce angular wind disturbance, so the influence of wind on an airship may be encapsulated as follows:

$$F_{w}=[\begin{array}{c} M{\dot{V}}_{a}+\Omega \times MV_{a}\\ O_{3\times 1} \end{array}],$$ (13)

where *M* contains the airship mass and added mass terms. Wind forces disturb the airship total velocity, angle of attack, and sideslip angle. Hence, its modified form can be represented by $V_{t}=\sqrt{u_{a}^{2}+v_{a}^{2}+w_{a}^{2}},\alpha =\tan^{-1}\left(w_{a}/u_{a}\right),\beta =\sin^{-1}\left(v_{a}/V_{t}\right)$.

The UETT airship flight is intended to be a low-altitude flight over around 300–400 feet of altitude because the airship will be used for low-altitude monitoring tasks. According to the military references [32], for low-altitude flights (below 1750 feet) and small aircraft, the turbulence scale length for clear air turbulence can be approximated by the following relations:

$$L_{w}=h,$$ (14)

$$L_{u}=L_{v}=\frac{h}{{\left(0.177+0.000823h\right)}^{1.2}},$$ (15)

where *h* is the altitude in feet. Typically, Taxila and Islamabad areas of Pakistan are marginal wind speed areas. In these areas, the wind speed varies between 3 and 6.2 ms^-1^ at about 200–400 feet of altitude [33]. Therefore, the turbulence intensities can be given as follows:

$$\sigma_{w}=0.1W_{200-400},$$ (16)

$$\sigma_{u}=\sigma_{u}=\frac{1}{{\left(0.177+0.000823h\right)}^{0.4}}0.1W_{200-400},$$ (17)

where *W*_200−400_ is the wind speed (in feet/sec) at fifty meters of altitudes. Therefore, the value of *W*_200−400_ will be 17.7165 feet/sec.

**Assumption 1.** The derivation of the 6-DOF model considers an airship as a rigid body and ignores the aeroelastic effects. Further, it is considered that the CG point of the airship lies beneath the CV. However, the variations of CG and aeroelastic effects are treated as model uncertainties, estimated using a lumped uncertainties estimation approach.

**Assumption 2.** The mass matrix parameters *m*_*i*_ = *m*_*i*0_+Δ*m*_*i*_ (*i* = 1,2,…,16) are uncertain with a known part *a*_*i*0_ and an uncertain part Δ*m*_*i*_. The uncertain part is bounded by some upper bound ${\bar{m}}_{imax}$. The aerodynamic model coefficients *C*_*ij*_ = *C*_*ij*0_+*C*_*ij*Δ_ (*i* = *X*,*Y*,*Z*,*L*,*M*,*N*;*j* = 1,2,3,4) are uncertain, where *C*_*ij*0_ is a known part and *C*_*ij*Δ_ is an unknown part bounded by some upper bound ${\bar{C}}_{ijmax}$. The CG point {*a*_*x*_ = *a*_*x*0_+Δ*a*_*x*_, *a*_*z*_ = *a*_*z*0_+Δ*a*_*z*_} is uncertain with a known part {*a*_*x*0_, *a*_*z*0_} and an uncertain part {Δ*a*_*x*_, Δ*a*_*z*_}. The uncertain part is bounded by an upper bound $\left\{{\bar{a}}_{xmax},\;{\bar{a}}_{zmax}\right\}$.

**Assumption 3.** The airship is equipped with GPS and IMU and pressure measuring sensors from which its current position and attitude estimates are made. This assumption is realistic because the available off-the-shelf solutions perform sensor fusion using nonlinear estimators and provide the estimates of UAV position and attitude. According to the first assumption, the CG point lies beneath the CV. So, it is further assumed that the gondola and payload location constrain the airship roll angle and pitch angle to the limits {$|\phi |<\frac{\pi }{2};|\theta |<\frac{\pi }{2}$}, ensuring that the rotation matrix remains nonsingular.

**Assumption 4.** The airship is equipped with a pitot tube sensor that provides the measurement of its speed *V*_*t*_.

## 3. Unscented Kalman filter design for airship model uncertainties and wind disturbance estimation

In engineering, filtering and estimation are inescapable tools that are almost a part of every control and signal processing problem. The estimators play a key role in finding the true state information from the noisy sensor measurements [34–37]. If the problem lies in the linear domain, then the best optimal minimum least square estimator is the Kalman filter. In theoretical terms, the Kalman filter is an estimator for the linear-quadratic problem where it is desired to estimate the instantaneous state of a linear system perturbed with white noise. This is done by utilizing measurements that are linearly related to the system’s states but corrupted with white noise. Practically, it is one of the important discoveries in statistical estimation theory. It enabled engineers to solve many problems that were not possible without it.

However, for nonlinear dynamical systems, Schmidt has discovered the improved version of the Kalman filter, i.e., Extended Kalman Filter (EKF), a recursive filter that linearizes the system on every sampling instant and applies linear Kalman filter equations. EKF has widespread utility in many important real-world applications, such as the control of ships, aircraft, and spacecraft. Although the utility of EKF in practical applications cannot be denied, its performance may be degraded in some cases where strong nonlinearities exist or the equations for the nonlinear dynamical system are not well known. In such cases, it suffers from implementation issues due to the calculation of the Jacobian matrix.

In 1997, Julier and Ohlmen introduced the basic equations for the UKF that address the deficiencies of EKF and avoid the calculation of the Jacobian matrix [38]. Contrary to the EKF, UKF accurately captures the mean and covariance up to the second order of Taylor series expansion for any nonlinear system. UKF is based on the deterministic sampling approach where a few sample points are carefully chosen, called sigma points. The state distribution is again assumed as Gaussian random variables. The carefully selected sigma points are passed through Unscented Transformation (UT). In UT, the sigma points are passed through the actual nonlinear system model and transformed points are obtained. The mean and covariance of the transformed points are calculated. This mean and covariance represent the true mean and covariance of the system.

**Fig 2:**
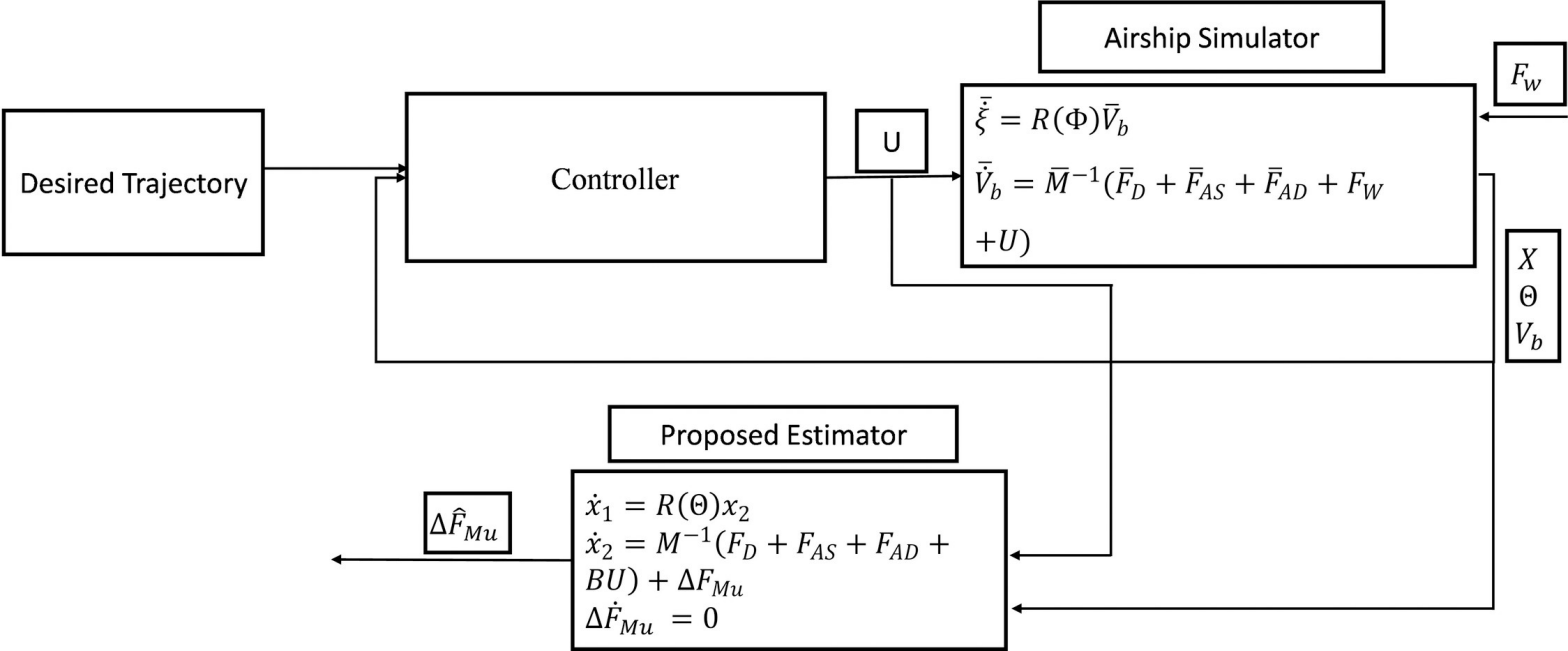
Generalized flowchart for the estimator.

UKF algorithm can be divided into two steps: the prediction step and the correction step. In both steps, the same UT is used for the system state model and measurement model. Fig 2. shows the basic flowchart for the Kalman filter algorithm. It highlights the basic governing steps for the Kalman filter and its variants. The Kalman filter algorithm and its variants have been utilized to estimate system measurable and unmeasurable states, unknown parameters, disturbances, and system faults. For such applications, the system is represented in state-space form. The unknown parameters of interest, such as unknown model parameters, disturbances, and system faults, are represented as additional state variables. In such a situation, it estimates both system states and additional unknown variables of interest.

For airship states, aerodynamic model parameters estimation, and aerodynamic model estimation, few contributions can be found in the literature as mentioned earlier. The airship aerodynamic model parameters are estimated using UKF; however, this approach is computationally overburdened and cannot be preferred as an online estimation solution. To overcome its deficiencies, the estimation strategy for the complete aerodynamic model (instead of its individual parameters) is also proposed using only six augmented state variables. The suggested approach is computationally efficient and can provide an online estimate of the airship aerodynamic model. However, it is based on the assumption that airship mass matrix and dynamic and aerostatic force vectors are known. Moreover, airship parameters are not varying during the complete flight operation.

This article proposes a general estimation solution without considering such an assumption. An estimation framework is presented where airship model uncertainties, airship states, and wind disturbance are estimated by introducing only six auxiliary state variables. The proposed work is intended to be computationally less intensive and it aims to provide a comprehensive online estimation solution for implementing a nonlinear controller. A lumped estimation approach based on UKF is proposed where changes in the airship model due to model uncertainties and wind disturbances are combined in a single vector. First, the airship known model (model with nominal parameters) is separated from the uncertain part (perturbed part). Then, the complete airship model is represented in terms of a known part and an uncertain part. For the uncertain part, a lumped uncertainty vector is introduced. For the known part of the airship model, the following assumption is introduced.

**Assumption 5.** The only known part of the airship model as defined in Assumption 2 is actually available. While the vector of wind forces *F*_*w*_ is unknown, it is bounded by a scalar *F*_*wmax*_>0, i.e., ‖*F*_*w*_‖<*F*_*wmax*_.

By utilizing Assumption 5, the known part of the airship model can be formulated as follows:

$${\dot{\hat{x}}}_{1}=R(\Theta ){\hat{x}}_{2},$$ (18)

$${\dot{\hat{x}}}_{2}=M^{-1}(F_{D}+F_{AS}+F_{AD}+BU),$$ (19)

where the terms *M*, *F*_*D*_, *F*_*AS*_, *F*_*AD*_, and B are defined in (5–8), respectively, with known values (nominal values) as given in Assumption 2. In lumped approach, a unified term (lumped term) in the known airship model (18–19) covers the deficiencies in the model. The modified model incorporating model uncertainties can be represented by the following equations:

$${\dot{x}}_{1}=R(\Theta )x_{2},$$ (20)

$${\dot{x}}_{2}=M^{-1}(F_{D}+F_{AS}+F_{AD}+BU)+\Delta F_{Mu},$$ (21)

where

$$\Delta F_{Mu}={\bar{M}}^{-1}({\bar{F}}_{D}+{\bar{F}}_{AS}+{\bar{F}}_{AD}+F_{W}+BU)-M^{-1}(F_{D}+F_{AS}+F_{AD}+BU).$$ (22)

For the application of the UKF algorithm, the airship model defined in (16–17) is represented in nonlinear state-space form, and six additional state variables are introduced for lumped model uncertainty vector.

Let

$$F=F_{D}+F_{AS}+F_{AD}+BU.$$ (23)

Moreover, the lumped uncertainty vector is defined as follows:

$$\Delta F_{Mu}=[\Delta F_{u}\;\Delta F_{v}\;\Delta F_{w}\;\Delta F_{p}\;\Delta F_{q}\;\Delta F_{r}].$$ (24)

The uncertainty vector estimates the total effect on airship body axes’ linear and angular acceleration due to the variations that occur in the airship model. Δ*F*_*u*_, Δ*F*_*v*_, and Δ*F*_*w*_ represent the effects on the forward, sway, and vertical acceleration, while the effects on the roll, pitch, and yaw acceleration are represented by Δ*F*_*p*_, Δ*F*_*q*_, and Δ*F*_*r*_, respectively. The modified model can be formulated as follows:

$$\left[\begin{array}{c} {\dot{x}}_{1}\\ {\dot{x}}_{2}\\ \Delta {\dot{F}}_{Mu} \end{array}\right]=[\begin{array}{c} R(\Theta )x_{2}\\ M^{-1}F+\Delta F_{Mu}\\ O_{6\times 1} \end{array}],$$ (25)

where *O*_6×1_ is the matrix with all zero elements. The new model state vector consists of eighteen state elements defined as follows:

$$X=[P\;\Phi \;\nu \;\Omega \;\Delta F_{Mu}].$$ (26)

According to Assumption 3, the state measurement vector can be defined as follows:

$$Y=CX=[I_{12\times 12}\;O_{12\times 6}]X.$$ (27)

Eq (21) represents the continuous-time state-space representation of the airship model. Its compact representation is given in Eq (24).


$$\dot{X}=f(X,u).$$ (28)

For the discrete-time UKF algorithm implementation, explicit first-order Euler integration is performed in (24). Moreover, it is augmented with process and measurement noise. The discrete-time representation of the model (28) is given as follows:

$$X_{k+1}=IX_{k}+T_{s}f(X_{k},u_{k})+W_{p},$$ (29)

$$Y=CX_{k}+W_{m},$$ (30)

where *X*_*k*_ represents the discrete-time state vector, *T*_*s*_ is the sampling time, *W*_*p*_ is the process noise vector, and *W*_*m*_ is the measurement noise vector. *W*_*p*_ and *W*_*m*_ have white noise with zero mean. The process noise and measurement noise covariance matrixes are represented by *Q* and *R*, respectively.

UKF algorithm at each sampling time is summarized as follows.

### Prediction

The sigma point at time step *k* is selected:

$${\hat{\chi }}_{k-1}^{\left(0\right)}={\hat{X}}_{k-1},$$ (31)

$${\hat{\chi }}_{k-1}^{\left(j\right)}=\left[{\hat{X}}_{k-1}+{\left(\sqrt{NP_{k-1}}\right)}_{j}{\hat{X}}_{k-1}-{\left(\sqrt{NP_{k-1}}\right)}_{j}\right]\;j=1,\ldots ,N$$ (32)

UT is applied to the state function:

$${\hat{\chi }}_{k}^{\left(j\right)}=f({\hat{\chi }}_{k-1}^{\left(j\right)},u_{k}),$$ (33)

$${\hat{X}}_{k}=\sum_{j=0}^{2N}W_{N}^{j}{\hat{\chi }}_{k}^{j}\;j=1,\ldots ,N,$$ (34)

$$P_{k}=\sum_{j=0}^{2N}W_{N}^{j}{\left({\hat{\chi }}_{k}^{\left(j\right)}-{\hat{X}}_{k}\right)}^{T}({\hat{\chi }}_{k}^{\left(j\right)}-{\hat{X}}_{k})+Q.$$ (35)

### Correction

UT is applied to the measurement function:

$${\hat{\Upsilon }}_{k}^{j}=h({\hat{\chi }}_{k}^{\left(j\right)},u_{k}),$$ (36)

$${\hat{Y}}_{k}=\sum_{j=0}^{2N}W_{N}^{j}{\hat{\Upsilon }}_{k}^{j},$$ (37)

$$P_{y}=\sum_{j=0}^{2N}W_{c}^{j}{\left({\hat{\Upsilon }}_{k}^{j}-{\hat{Y}}_{k}\right)}^{T}\left({\hat{\Upsilon }}_{k}^{j}-{\hat{Y}}_{k}\right)+R,$$ (38)

$$W_{c}^{j}=\frac{1}{2N}\;j=1,2,\ldots ,2N.$$ (39)

The cross-correlation is calculated:

$$P_{xy}=\frac{1}{2N}\sum_{j=0}^{2N}\left({\hat{\chi }}_{k}^{\left(j\right)}-{\hat{X}}_{k})({\hat{\Upsilon }}_{k}^{j}-{\hat{Y}}_{k}\right).$$ (40)

The Kalman gain, corrected state vector, and error covariance matrix are calculated:

$$K_{k}=P_{xy}P_{y}^{-1},$$ (41)

$${\hat{X}}_{k+1}={\hat{X}}_{k}+K(Y_{k}-{\hat{Y}}_{k}),$$ (42)

$$P_{k+1}=P_{k}-K_{k}P_{y}K_{k}^{T},$$ (43)

where *P*_*k*_ is the state error covariance matrix. In the prediction phase, the first step is to select sigma points, which are actually spread around the current state value. The number of sigma points depends on the total number of states. Then, these points are passed through UT. In UT, sigma points are subjected to the nonlinear airship model and transformed points are obtained. Further, the weighted mean and covariance of transformed points are calculated. In the correction step, first, the transformed sigma points are passed through UT, where they are subjected to the measurement model. Then, the current output measurement covariance is calculated. Cross-covariance of data is calculated using transformed sigma points obtained through state model, measurement model estimated state, and estimated measurement. In the last step, Kalman gain is calculated and utilized for the correction of state and state error covariance.

## 4. Results and discussion

**Fig 3:**
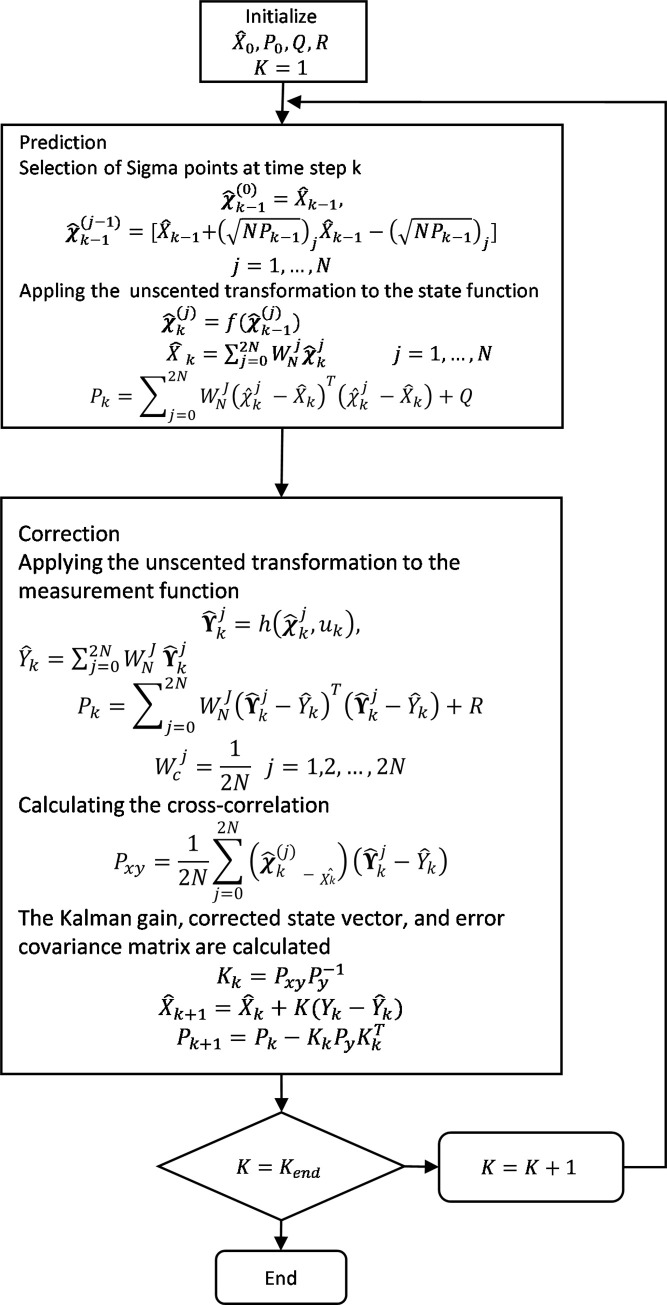
Block diagram of the simulation environment involving the estimation of the proposed estimator.

The performance of the proposed estimator has been verified by developing the simulation environment for the experimental UETT airship under the autonomous UAV development project for environmental monitoring tasks. A nonlinear 6-DOF simulation model of the airship is developed in MATLAB/Simulink R2019b with a variable step R-K (Runga-Kutta) method on a computer having a CPU frequency of 2.5 GHz and 8 GB of RAM. For the simulation, 0.002 s sampling time is used. Fig 3 shows the block diagram of the proposed algorithm to estimate the required parameters. The airship simulator uses the nonlinear dynamic equations of the airship model for UETT airship [39, 40]. Mass matrix parameters for the airship are given in S1 Appendix.

For comparative study, EKF is designed for the same application. EKF requires the computation of the Jacobian matrix. Therefore, the analytical expressions for the Jacobian matrix are established and given in S2 Appendix. EKF algorithm is based on the same prediction and correction steps; however, it goes through the linearization step following the flow illustrated in Fig 2.

For performance evaluation, three different cases have been considered. The first case deals with the estimation of the uncertainty vector defined in (24). Estimation is performed subject to the variation in the airship aerodynamic model. The second case is about the estimation of mass matrix parameters. The third case discusses wind disturbances as applied to the airship during its aerodynamic flight. For all cases, a controlled flight is considered. A change in the parameters of an aerodynamic model and a mass matrix is introduced during the flight.

### 4.1. Airship aerodynamic model estimation

**Fig 4:**
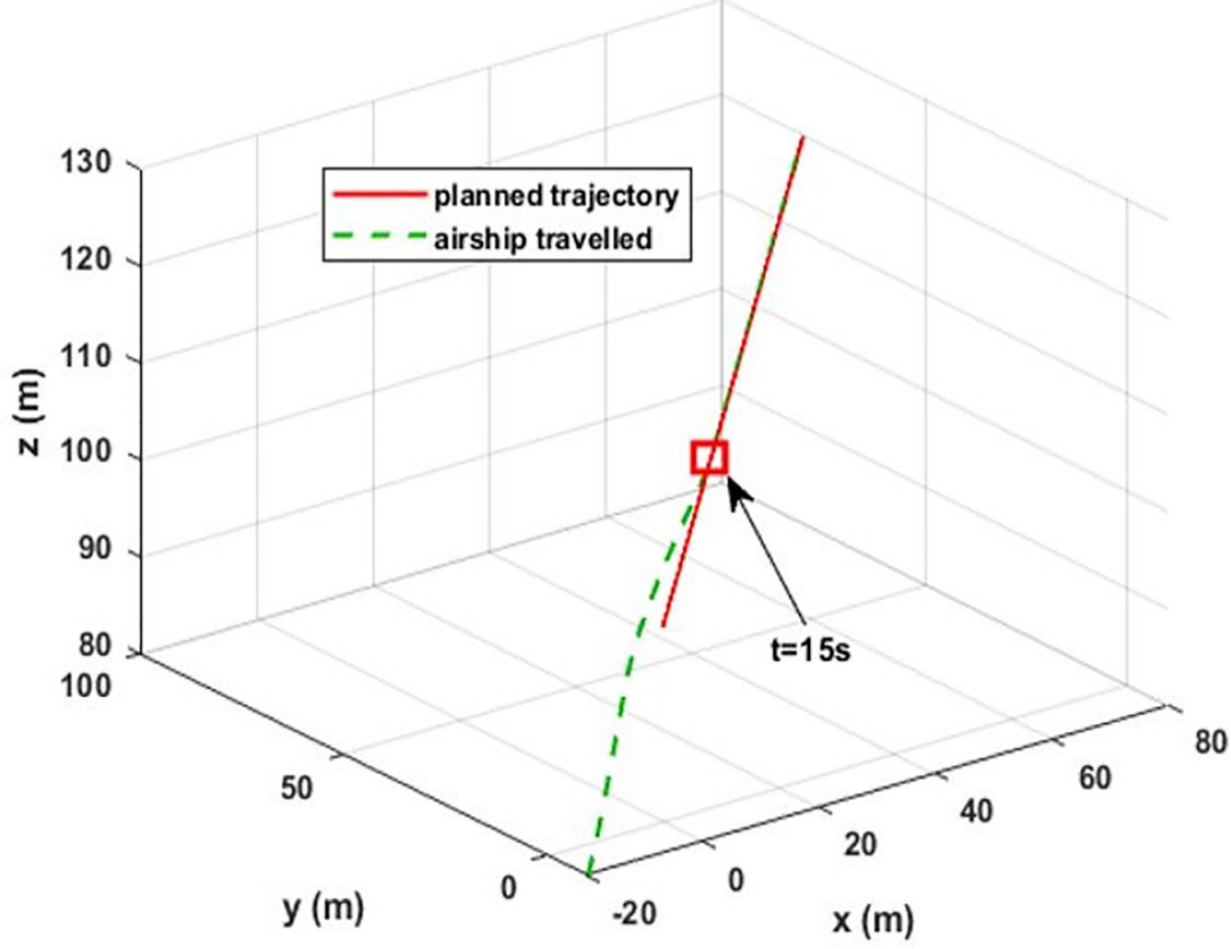
Airship-controlled flight. The dashed green line indicates the actual path traveled by the airship, and the red line shows the desired trajectory. At the 15 sec point, the airship reaches the desired trajectory and starts a steady-state flight. The start of steady-state flight is indicated by a red rectangle.

The airship-controlled flight is illustrated in Fig 4. The airship moves along the predefined three-dimensional straight-line trajectory while executing controlled aerodynamic flight. In order to get the transient flight state, different initial conditions for the desired trajectory and airship simulator are considered. Large variations in all the states can be seen in the transient part of the flight. In a steady state, the airship follows the desired trajectory and starts executing the smooth flight by stabilizing all the states. The desired trajectory starts from the point [45 m, 60 m, −80 m]. The airship simulator is initialized with P_0_, Φ_0_, *υ*_0_, Ω_0_ values as given in Table 1. The initial conditions used for the estimator are given in Table 2.

**Table 1:** Initial conditions for airship simulator.

State	Symbol	Value	Units
Position	P_0_	[−20, −10, −80]	m
Attitudes	Φ_0_	[0, 0, 0.5]	rad
Linear velocities	*υ*_0_	[0.4, 0.2, 0.1]	ms^-1^
Angular velocities	Ω_0_	[0, 0, 0.001]	rads^-1^

**Table 2:** Initial conditions for estimatior.

State	Symbol	Value	Units
Position	P_0_	[−24, −12, −96]	m
Attitudes	Φ_0_	[0.2, 0.2, 0.6]	rad
Linear velocities	*υ*_0_	[0.48, 0.24, 0.12]	ms^-1^
Angular velocities	Ω_0_	[0.2, 0.2, 0.0012]	rads^-1^
Uncertainty vector	Δ*F*_*Mu*0_	[0 0 0.2 0 0 0].	

Generally, the accuracy of the initial values facilitates the convergence of a system. The best initial values give the best convergence. In our case, to give an allowance for inaccurate initial values, we have tested our algorithm for a change of 20% in the accurate initial values.

For the present case, it is assumed that airship mass matrix parameters remain unchanged and wind disturbances are not acting on the airship. So, we can say that $\bar{M}=M,\;{\bar{F}}_{D}{=F}_{D},\;{\bar{F}}_{AS}=F_{AS}$ and *F*_*W*_ = 0. The lumped uncertainty vector is simplified to $\Delta F_{Mu}=M^{-1}({\bar{F}}_{AD}-F_{AD})$. It indicates that the lumped uncertainty vector encapsulates the difference between the actual aerodynamic forces acting on the airship and the aerodynamic model used in the estimator design. After 15 seconds of flight, a change *δ*, in the aerodynamic force vector ${\bar{F}}_{AD}$, is introduced such that ${\bar{F}}_{AD}=F_{AD}+\delta$ where *δ* is a six-dimensional vector and its value in simulation is selected as given in (27).

We have used the analytical expression for the aerodynamic modeling equation as given in [27]. The comparative study of the analytical model with Gomez’s wind tunnel model shows that the analytical aerodynamic modeling equations have 20% error in the side force coefficient, 10% error in the rolling moment coefficient, and 25% error in the yaw moment coefficient. To be on the safe side, we have considered a 30% change in the steady-state aerodynamic forces and torques.


δ=[−.45.6.09.3.63.6]. (44)

**Fig 5:**
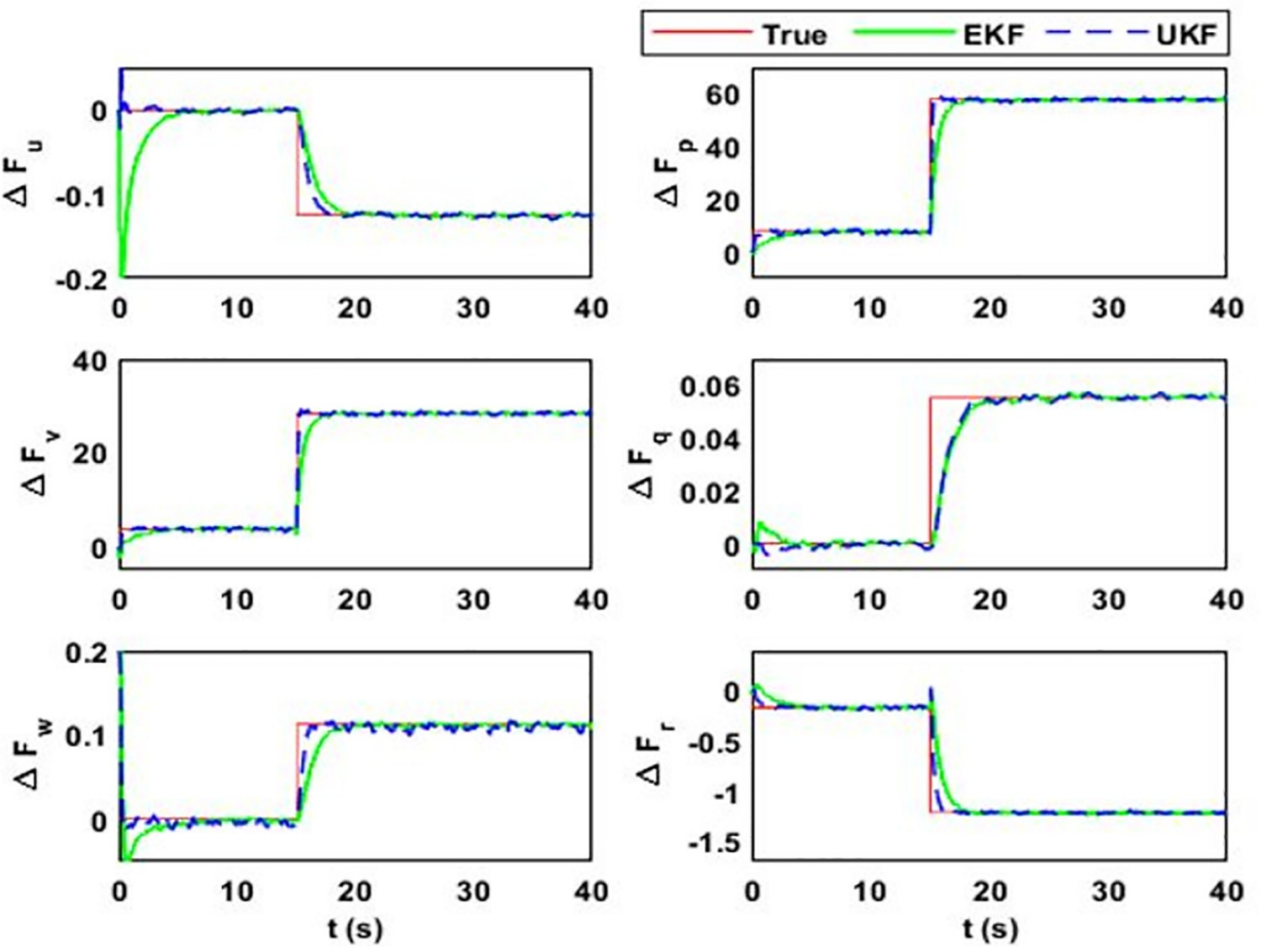
Estimation of model uncertainty and disturbance vector. In this case, it estimates the total effect on airship body axes linear and angular acceleration due to the variations that occur in the airship aerodynamic model: (i) Δ*F*_*u*_; (ii) Δ*F*_*p*_; (iii) Δ*F*_*v*_; (iv) Δ*F*_*q*_; (v) Δ*F*_*w*_; (vi) Δ*F*_*r*_.

**Fig 6:**
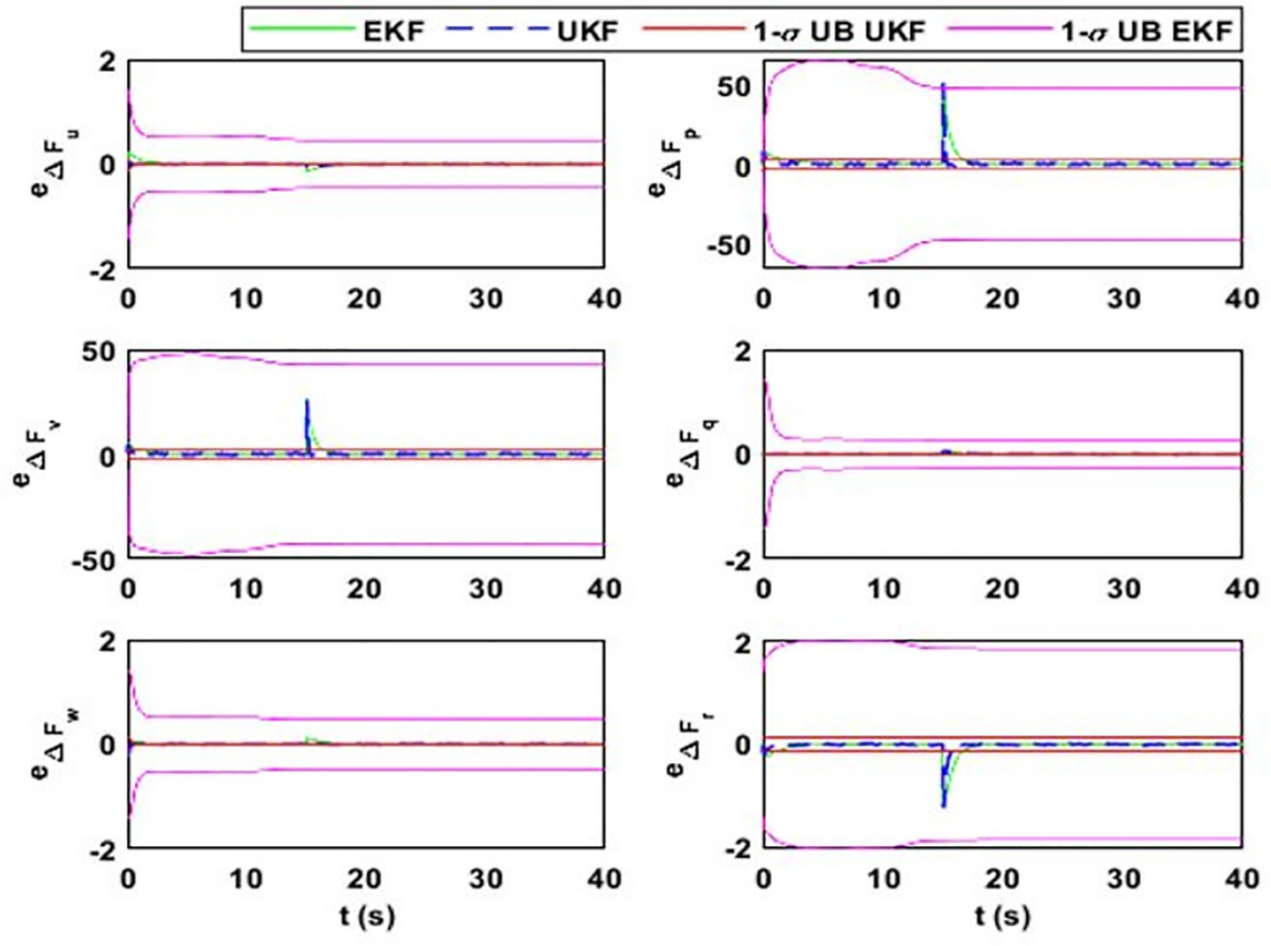
Estimation error and error bounds for lumped uncertainty vector showing the error and sigma bounds corresponding to (i) Δ*F*_*u*_; (ii) Δ*F*_*p*_; (iii) Δ*F*_*v*_; (iv) Δ*F*_*q*_; (v) Δ*F*_*w*_; (vi) Δ*F*_*r*_.

After 15 seconds of flight, the estimator estimates the change Δ*F*_*Mu*_ = *M*^−1^(*δ*). The estimation results for the uncertainty vector are summarized in Fig 5. The value of Δ*F*_*Mu*_ before 15 seconds is zero because ${\bar{F}}_{AD}=F_{AD}$, where ${\bar{F}}_{AD}$ is the aerodynamic model used in the simulator design and *F*_*AD*_ is in the estimator design. However, the simulator model observes *δ* change that alters the model uncertainty vector. Fig 6 shows the estimation error and error bounds for the estimated states and uncertainty vector.

### 4.2. Mass matrix parameter variations

In this case, it is considered that the airship aerodynamic model is known while its airship mass matrix suffers from parameter variations. Moreover, wind disturbances are not acting on the airship. Hence, the model uncertainty vector will be $\Delta F_{Mu}={\bar{M}}^{-1}({\bar{F}}_{D}+{\bar{F}}_{AS}+F_{AD}+BU)-M^{-1}(F_{D}+F_{AS}+F_{AD}+BU)$. The changes in mass matrix parameters are actually unavoidable in a real flight. During the airship flight for controlling buoyancy, the air from the air ballonet is charged in and out, which causes variation in an airship mass, and also a small amount of helium gas leakage is inevitable. These variations also change the CG point. The performance of an autonomous flight controller can be improved if the model uncertainty information is calculated online and provided to the controller.

**Fig 7:**
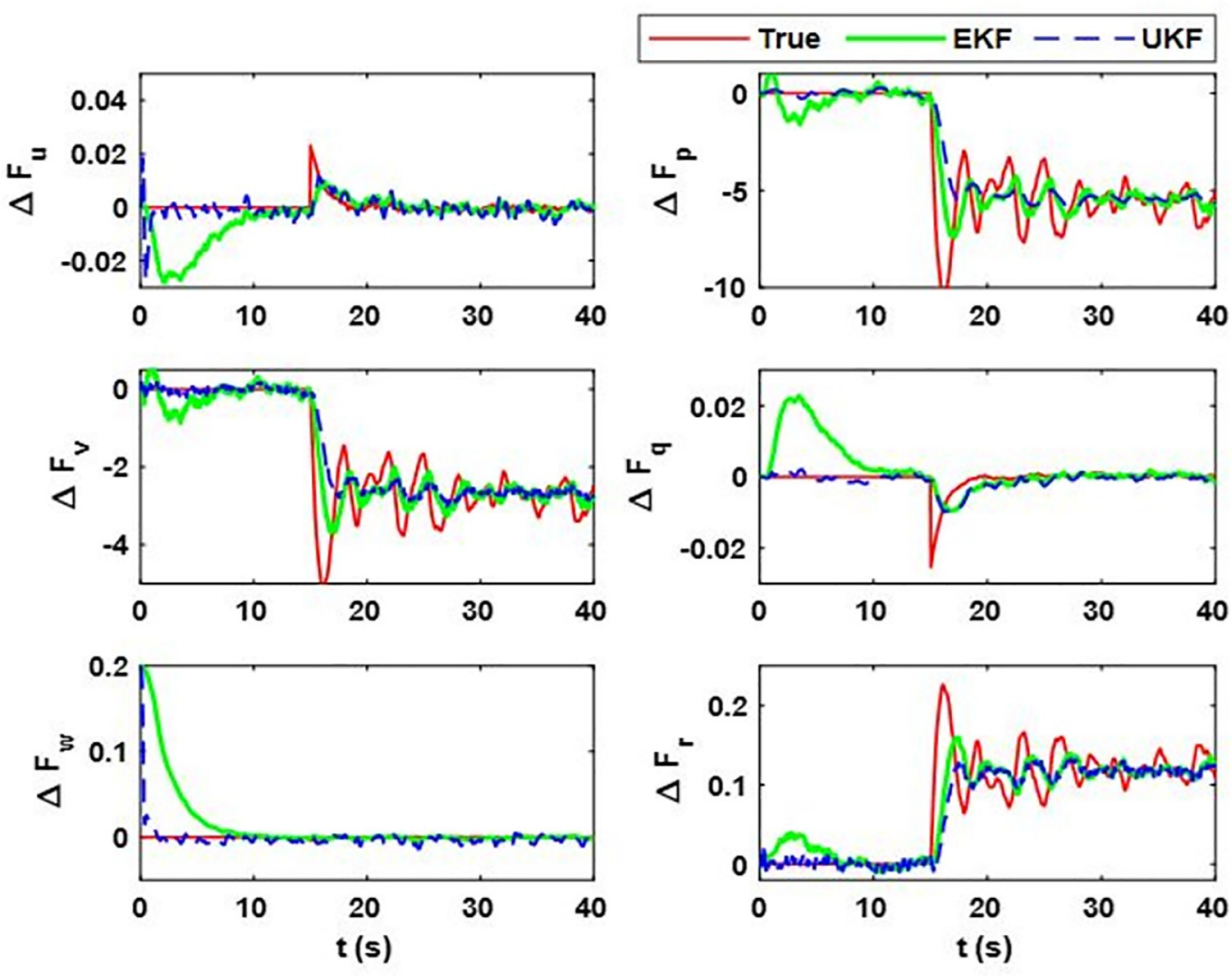
Estimation of model uncertainty and disturbance vector. In this case, it estimates the total effect on airship body axes’ linear and angular acceleration due to the variations that occur in the airship mass matrix. (i) Δ*F*_*u*_; (ii) Δ*F*_*p*_; (iii) Δ*F*_*v*_; (iv) Δ*F*_*q*_; (v) Δ*F*_*w*_; (vi) Δ*F*_*r*_.

**Fig 8:**
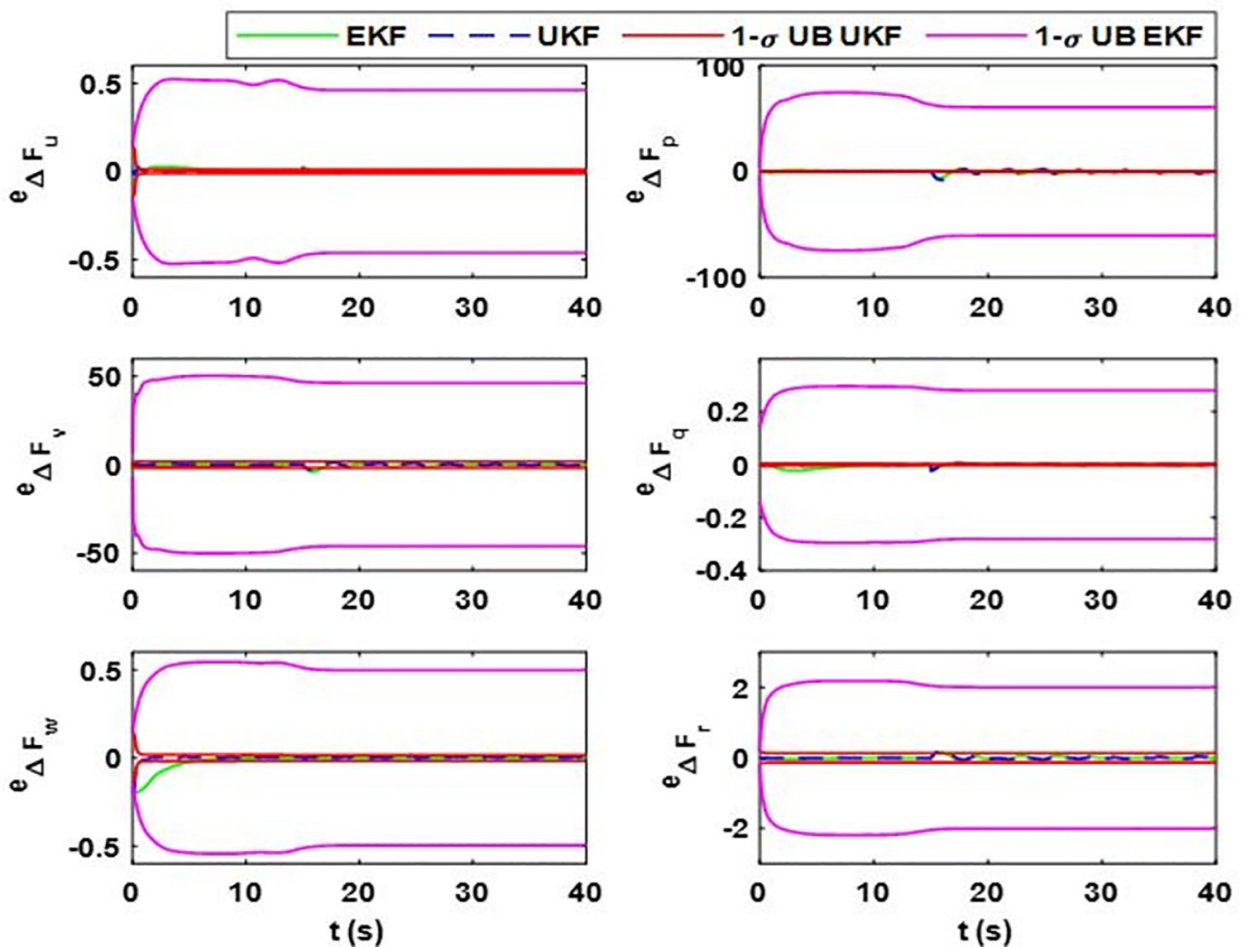
Estimation error and error bounds for lumped uncertainty vector showing the error and sigma bounds corresponding to (i) Δ*F*_*u*_; (ii) Δ*F*_*p*_; (iii) Δ*F*_*v*_; (iv) Δ*F*_*q*_; (v) Δ*F*_*w*_; (vi) Δ*F*_*r*_.

During the flight, the random error in mass matrix parameters can happen; that is, why the researchers have used a change of 20% in airship model parameters [41]. For further overconfidence of our proposed techniques, we have used 30% changes in mass matrix parameters after 15 seconds of the airship aerodynamic flight. The proposed estimation scheme is used to estimate the changes. Fig 7 shows the filter estimated model uncertainty vector. Moreover, their estimation error and uncertainty bounds are given in Fig 8.

### 4.3. Wind disturbance case

In order to evaluate the estimator performance subject to the wind disturbances, the following assumptions are made:

**Assumption 6.** Airship mass matrix parameters remain the same during the course of the flight.

**Assumption 7.** The aerodynamic model given in (7) is not considered in the estimator design because the aerodynamic forces depend on an airship’s relative velocity. This assumption will simplify the process of estimating wind forces along with aerodynamic forces instead of estimating them separately.

**Fig 9:**
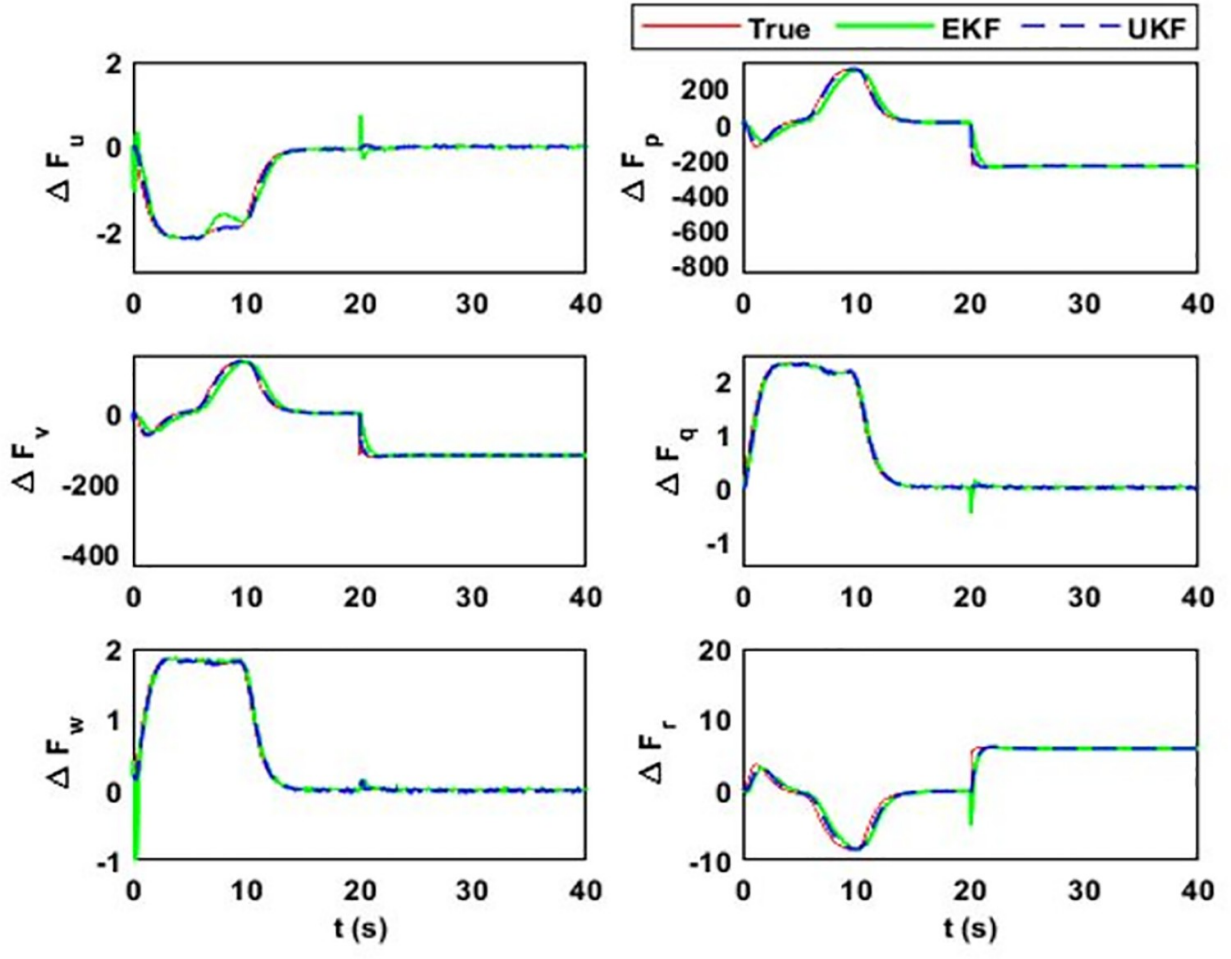
Estimation of model uncertainty and disturbance vector. In this case, the total effect on airship body axes linear and angular acceleration is estimated due to the aerodynamic model and wind disturbance corresponding to (i) Δ*F*_*u*_; (ii) Δ*F*_*p*_; (iii) Δ*F*_*v*_; (iv) Δ*F*_*q*_; (v) Δ*F*_*w*_; (vi) Δ*F*_*r*_.

**Fig 10:**
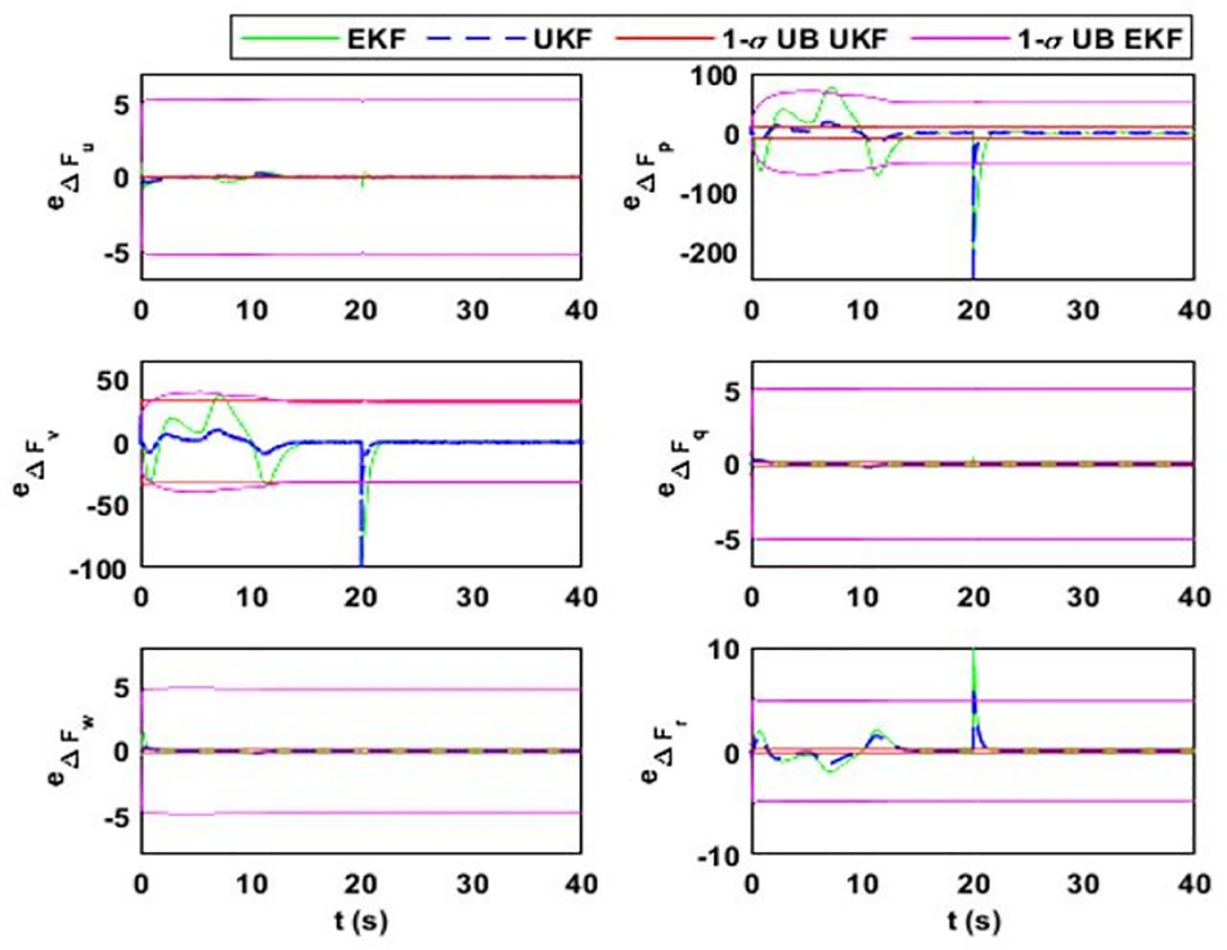
Estimation error and error bounds for lumped uncertainty vector, with the error and sigma bounds for (i) Δ*F*_*u*_; (ii) Δ*F*_*p*_; (iii) Δ*F*_*v*_; (iv) Δ*F*_*q*_; (v) Δ*F*_*w*_; (vi) Δ*F*_*r*_.

Hence, the lumped model uncertainty vector estimated by the estimator will reduce to $\Delta F_{Mu}=M^{-1}(F_{D}+F_{AS}+F_{AD}+F_{w}+BU)-M^{-1}(F_{D}+F_{AS}+BU)=M^{-1}(F_{AD}+F_{w})$, since, in this case, $\bar{M}=M,\;{\bar{F}}_{D}{=F}_{D},\;{\bar{F}}_{AS}=F_{AS}$. The same autonomous flight is considered, as discussed in case 1. Wind disturbances are applied after 20 seconds of the airship flight and the estimation results for the model uncertainty vector are illustrated in Fig 9 and their respective error bounds are given in Fig 10.

Fig 9 shows the estimation of the uncertainty vector under variations in aerodynamic forces and wind disturbances. As it is assumed that wind acts on the airship after 20 seconds of flight, the uncertainty vector initially captures only the aerodynamic forces. Fig 9 (a) shows the drag force acting on the airship. The initial value of the drag force is high due to the high initial value of the airship forward velocity component. When the airship starts following the desired trajectory, then its forward velocity and drag force stabilize to the steady-state values. Initially, the airship is at a different position from the planned trajectory and the controller tries to maneuver it toward the planned trajectory. Consequently, the airship experiences side and lift aerodynamic forces.

Although from Fig 4, in steady state, it can be seen that airship executes rectilinear motion in 3D space, it has a positive value of only forward velocity component. This is because, in a steady state, the airship is heading in the direction of motion with a small pitch and has zero sideslip and angle of attack. After 20 seconds of flight, wind disturbance is applied from inertial ***X***_***e***_-axis in ***X***_***e***_***Y***_***e***_ plane. It affects the airship belly at the left side and tries to push the airship out of the desired path. This effect can be seen in Fig 10, where the changes in the side force, rolling torque, and yaw torque are produced by wind.

### 4.4. Performance analysis and comparative study

In all cases, we can see that the proposed approach is able to estimate the model uncertainties and external disturbances applied to the system. Model uncertainties can be due to deficiencies in modeling, incorrect model parameters, parameter variations, or unmodelled dynamics. In the cases presented above, we have discussed all of these uncertainties like the parameter variation of the mass matrix (in case 2), aerodynamic model (in case 1), and unmodelled dynamics (in case 3), where the aerodynamic model is assumed to be unknown. In this section, to ascertain the quality of estimates, a kind of error analysis is performed. To obtain the confidence on estimates, a comparative study is conducted where the proposed estimator’s results are compared with the EKF.

Although the plots in Figs 5, 7, and 9 indicate that the proposed estimation method has a small estimation error; however, statistical analysis has to be performed to quantify it. The error between the true values and the estimated ones is calculated and they are evaluated using 1*σ* uncertainty bounds, where *σ* is the standard deviation of error. It refers to the amount of variability in a given set of data: whether the data points are all clustered together or are heavily spread out. For Gaussian distributions, the statistical rule says that about 68% of the data should lie within one standard deviation about the mean value, 95% within two standard deviations, and 99.7% within three standard deviations [42].

In this work, the error analysis is performed for each state and for all the cases. the mean estimation error (*M*_*e*_) and standard deviation (*σ*) are calculated for the states and model uncertainty vector. Results are summarized in Tables 3–5. The criteria of judgment for *M*_*e*_ are as follows [43]:

1. *M*_*e*_ should be less than *ε*, where *ε* is an upper bound specified by the designer of the controller for an airship.
2. It should be centered on zero.

**Table 3:** Case 1: Mean estimation error for all states.

State	EKF		UKF	
	*M*_*e*_	*σ*	*M*_*e*_	*σ*
**Δ*F***_***u***_	4.5e-3	0.48	-1.7e-3	0.011
**Δ*F***_***v***_	0.4687	43.6	0.1346	1.906
**Δ*F***_***w***_	6.2e-3	0.49	4.2e-3	0.017
**Δ*F***_***p***_	0.9830	51.3	0.2751	2.396
**Δ*F***_***q***_	5e-4	0.29	1.3e-4	0.002
**Δ*F***_***r***_	-3.6e-2	1.92	-1.56e-2	0.136

**Table 4:** Case 2: Mean estimation error for all states.

State	EKF		UKF	
	*M*_*e*_	*σ*	*M*_*e*_	*σ*
**Δ*F***_***u***_	1.5e-3	0.46	6e-4	0.011
**Δ*F***_***v***_	-7.7e-3	46.5	-5.06e-3	1.723
**Δ*F***_***w***_	-5e-3	0.49	2.3e-3	0.017
**Δ*F***_***p***_	-1.8e-2	61.9	-1.06e-2	0.331
**Δ*F***_***q***_	-1.1e-3	0.28	4e-4	0.002
**Δ*F***_***r***_	1e-4	2.01	2.5e-3	0.134

**Table 5:** Case 3: Mean estimation error for all states.

State	EKF		UKF	
	*M*_*e*_	*σ*	*M*_*e*_	*σ*
**Δ*F***_***u***_	-5.1e-2	5.17	4.1e-3	0.052
**Δ*F***_***v***_	-5.4	33.2	-1.13	32.77
**Δ*F***_***w***_	-1.1e-2	4.81	5.1e-3	0.095
**Δ*F***_***p***_	-11.09	53.9	-2.1	9.33
**Δ*F***_***q***_	2.4e-2	5.08	-2.3e-2	0.094
**Δ*F***_***r***_	0.29	4.86	0.17	0.138

The results of the tables show that both estimators obey the minimum judgment criteria, thus indicating the reliability of the estimates.

For case 1, the estimation error and uncertainty bounds for the airship uncertainty and disturbance vector are given in Fig 6. From the figure, it can be seen that both estimators perform adequately. The filter’s performance in the transient state is quantified from the figure where convergence time for each estimate can be noted. Moreover, from the data in the table, estimator performance for a steady state is quantified. The data in the table shows that *M*_*e*_ values for the airship states are close to zero.

As given in (17), side velocity (Δ*F*_*v*_) and roll rate uncertainty (Δ*F*_*p*_) terms have deviations in errors. The EKF uncertainty bounds for Δ*F*_*v*_ and Δ*F*_*p*_ are 43.6 ms^-2^ and 51.3 rads^-2^, respectively. However, these values are 1.9 ms^-2^ and 2.3 rads^-2^, respectively, for UKF. It is because EKF estimation accuracy for side velocity and roll rate uncertainty terms are low compared to UKF. The large standard deviation in these terms means that large estimation error may occur with the probability of 0.68. The estimation error in Δ*F*_*v*_ can reach up to 43.6 ms^-2^ and for Δ*F*_*p*_, it can rise up to 51.3 rads^-2^ with a probability of 0.68. However, for UKF, the estimation error for both terms can rise up to 1.15 ms^-2^ and 1.41 rads^-2,^ respectively. It is because the UKF does not rely on the first-order linear approximation of the system. Also, it propagates the true mean and covariance.

EKF linearizes the system based on the current state estimates. If the initial state estimate is away from the true value, then the transient response of the filter is affected. Apart from the steady-state estimation accuracy, EKF converges late to the true value while UKF takes less time for convergence. To quantify the convergence time, the uncertainty term related to the vertical velocity is initialized with a 20% error, affecting the estimate of vertical velocity for EKF. EKF converges to the true estimate after 10 seconds and UKF converges after 1 second. The other notable figures in the table are related to the pitch rate. The analysis shows that for both transient and steady-state cases, UKF performs better than EKF.

Fig 7 shows the estimation error and uncertainty bounds for case 2, from which the assessment for transient response can be made. For the quantitative analysis of the steady-state response, the data for mean estimation error and the steady-state value of uncertainty bounds are given in Table 4. The plots show that the estimation error for the last two cases is more than that of case 1. This is because in case 2, the change in mass matrix parameters induces high-frequency changes in the uncertainty vector, for which it becomes difficult for both EKF and UKF to detect the changes. Actually, in the simulation, we have taken the worst-case scenario where a sudden change in mass matrix parameters is applied after 20 seconds of a simulation run. However, in a real-world scenario, mass matrix parameters vary smoothly. Although the estimation error in case 2 exists, it remains within uncertainty bound and obeys the minimum performance criteria and the estimation error for the steady state is less than that of the transient state.

From Fig 7, we can see that in the transient state, the estimation error for Δ*F*_*Mu*_ for EKF is more. In some cases, like for Δ*F*_*p*_, the error deviation is 1.61 rads^-2^; for Δ*F*_*v*_ and Δ*F*_*p*_, it is 46.6 ms^-2^ and 61.1 rads^-2^, respectively. It shows that for EKF, the estimation error for these terms can be large compared to the magnitude of the estimate. Moreover, for EKF, the state error covariance does not converge to zero and the probability of error remains high for steady states. In contrast, for UKF, we can see that the error deviations for Δ*F*_*v*_ and Δ*F*_*p*_ are 1.723 ms^-2^ and 0.331 rads^-2^, respectively, which is small compared to the EKF. Furthermore, UKF quickly converges to the true estimates with its estimation error within the minimum performance bounds.

In case 3, the estimation error for the airship states and uncertainty vector is around zero. This is because the estimation of uncertainty term for sway velocity is high. As in this case, after 20 seconds of flight, wind disturbance is applied that affects the lateral motion of the airship. A sudden change occurs in the sway velocity uncertainty (Δ*F*_*v*_) term that is approximately 200 ms^-2^, the same value as that of roll rate uncertainty term (Δ*F*_*p*_). The estimator detects the change after a delay, which induces a large estimation error. However, in a steady state, the estimator follows the true values. For UKF, the estimation error in the transient state is small compared to that in EKF.

From the above analysis, we can observe that in a transient state, UKF outperforms the EKF. In the steady state, large error bounds for EKF reveal that the estimator state error covariance does not converge to zero. This implies that in a steady state, EKF estimates have a high probability of a large estimation error.

For the analysis of the computational overhead of both estimators, few test scenarios are considered where the running time of both estimators is recorded. The observations indicate that the EKF method takes 60.38 milliseconds on average, while UKF takes 350.28 milliseconds per estimate. The computational intensiveness of the UKF algorithm is obvious because it has to handle all sigma points and performs fifteen Runge-Kutta integrations to propagate the sigma points. If Julier and Uhlmann’s method is used for reducing the number of sigma points [43], eight Runge-Kutta integrations will still be required. However, the EKF algorithm needs only one integration to complete the computation. For the airship, there are twelve actual state variables and six augmented ones, so a total of 18 states require the computation of a large Jacobian matrix. If the numerical computation method for calculating the Jacobean matrix is used, then EKF will require more computational time. Moreover, the analytical calculation of the Jacobian matrix is cumbersome, though it reduces the computational overhead. However, in the case of UKF, computation of the Jacobian matrix is not required.

From the above discussion, we can conclude that for an airship state and uncertainty estimation, the UKF algorithm performance is better than that of EKF but with the cost of computational overburden. As an airship is a slow-moving platform and has slow dynamics due to its large size, in our case, we prefer UKF over EKF estimation as the former provides more accuracy.

## 5. Conclusion

The strength of the proposed estimation method lies in the provision of a unified estimation solution for airship states, model uncertainties, and wind disturbances. The presented method suggests a lumped model uncertainties vector that introduces six augmented state variables for approximating model uncertainties and wind disturbances. First, the airship model is presented with a suitable assumption for accommodating system unmodelled dynamics and parameter variation. Then, an extended nonlinear state-space representation is given for the estimator design. From the information of a pitot tube sensor, airship position, attitudes, and airship known dynamic model, one can estimate the effects induced by wind forces instead of measuring wind velocities by costly instrumentation. The proposed method is easily realizable, cost-effective, and understandable.

Compared to intelligent estimation solutions, such as neural networks, the proposed method is a unified solution for estimating airship parameters. It does not assume the availability of all states and their derivatives. For all the three cases presented in the article, the comparison of UKF and EKF indicates a better performance of UKF for both transient and steady states. The analysis reveals that in a transient state, EKF converges late compared to the UKF. The magnitude of error bounds for EKF is very large in the steady state. It has a probability of 0.68 for a large estimation error. Overall, the results suggest that the proposed method can be utilized in airship autonomous flight with nonlinear controllers. The method can provide state and model uncertainty information that will enhance the controller performance in terms of its robustness.

## Supporting information

S1 Appendix Model parameters. (DOCX)

S2 Appendix Airship state Jacobian matrix for EKF. (DOCX)

S1 Nomenclature (DOCX)
