# Supplementary material for: Unscented Kalman filter for airship model uncertainties and wind disturbance estimation
Source: PLoS One. 2021 Nov 5;16(11):e0257849. doi: 10.1371/journal.pone.0257849 (PMC8570505; doi:10.1371/journal.pone.0257849)
Supplement: S1 Appendix — (DOCX) [file pone.0257849.s001.docx]

Appendix 1

Model PARAMETERS

| $\alpha$ | : | Angle of attack |
| --- | --- | --- |
| $\beta$ | : | Side slip angle |
| $V_{0}$ | : | Velocity of airship |
| $\rho$ | : | Air density |
| S | : | Airship total surface area |
| W | : | Weight of airship |
| B | : | Buoyancy force |
| M | : | Airship mass |
| $l$ | : | Airship length |
| $d$ | : | Airship diameter |
| $m_{1}=m_{x}$ | $=$ | $m-X_{\dot{u}}$ |
| $m_{2}=m_{y}$ | $=$ | $m-Y_{\dot{v}}$ |
| $m_{3}=m_{z}$ | $=$ | $m-Z_{\dot{w}}$ |
| $m_{5}=m_{15}$ | $=$ | ${ma}_{z}-X_{\dot{q}}$ |
| $m_{4}=m_{24}$ | $=$ | ${-ma}_{z}-Y_{\dot{p}}$ |
| $m_{6}=m_{26}$ | $=$ | ${ma}_{x}-Y_{\dot{r}}$ |
| $m_{7}=m_{35}$ | $=$ | $-{ma}_{x}-Z_{\dot{q}}$ |
| $m_{8}=m_{42}$ | $=$ | $-{ma}_{z}-L_{\dot{v}}$ |
| $m_{11}=m_{51}$ | $=$ | ${ma}_{z}-M_{\dot{u}}$ |
| $m_{12}=m_{53}$ | $=$ | ${-ma}_{x}-M_{\dot{w}}$ |
| $m_{14}=m_{62}$ | $=$ | ${ma}_{x}-N_{\dot{v}}$ |
| $m_{9}=J_{x}$ | $=$ | $I_{x}-L_{\dot{p}}$ |
| $m_{10}=-J_{xz}$ | $=$ | $I_{xz}-L_{\dot{r}}$ |
| $m_{13}=J_{y}$ | $=$ | $I_{y}-M_{\dot{q}}$ |
| $m_{15}{=-J}_{xz}$ | $=$ | $I_{xz}-L_{\dot{r}}$ |
| $m_{16}=J_{z}$ | $=$ | $I_{z}-N_{\dot{r}}$ |
| $Z_{\dot{w}}$ | $=$ | $Y_{\dot{v}}$ |
| $N_{\dot{r}}$ | $=$ | $M_{\dot{q}}$ |
| $\bar{m}$ | $=$ | $B/g$ |
| $\bar{I}_{y}$ | $=$ | $\frac{\bar{m}(l^{2}+d^{2})}{20}$ |
| $x_{cv}$ | $=$ | $a_{1}+\frac{3}{8}\left( a_{2}-a_{1} \right)$ |

$I_{x}, I_{y} and I_{z}$ are the moment of inertia about $ox$, $oy$, and $oz,$ respectively. $I_{xz}$ is the product of inertia about $oy$.

$X_{\dot{u}}$, $Y_{\dot{v}}$, $Z_{\dot{w}}$, $X_{\dot{q}}$, $Y_{\dot{p}}$, $Y_{\dot{r}}$, and $Z_{\dot{q}}$ are the virtual mass terms.

$L_{\dot{p}}$, $N_{\dot{p}}$, $M_{\dot{q}}$, $L_{\dot{r}}$, $L_{\dot{v}}$, $M_{\dot{u}}$, $M_{\dot{w}}$, $N_{\dot{v}}$, and $N_{\dot{r}}$ are the virtual inertia terms. Some virtual mass and inertia terms are disregarded and the rest of them can be calculated using the standard equations originally given by Lamb in [25].

$a_{x}$ and $a_{z}$ are the coordinates of CG with respect to CV.

$K_{1}$ is the Lamb’s ratio of inertia along the longitudinal axis $ox$.

$K_{2}$ is the Lamb’s ratio of inertia along the lateral axis $oy$.

$K^{'}$ is the Lamb’s ratio of inertia for rotation along the lateral axis $oy$.
