# Supplementary material for: Unscented Kalman filter for airship model uncertainties and wind disturbance estimation
Source: PLoS One. 2021 Nov 5;16(11):e0257849. doi: 10.1371/journal.pone.0257849 (PMC8570505; doi:10.1371/journal.pone.0257849)
Supplement: S2 Appendix — (DOCX) [file pone.0257849.s002.docx]

Appendix 2

Airship State Jacobian Matrix for EKF

$$A=\left[ \begin{matrix} I_{3\times3} & \sum_{i=1}^{3} \sum_{j=1}^{9} a_{ij} & O_{3\times6} \\ O_{3\times3} & \sum_{i=4}^{6} \sum_{j=1}^{9} a_{ij} & O_{3\times6} \\ O_{6\times3} & \sum_{i=7}^{12} \sum_{j=1}^{9} a_{ij} & M^{-1} \\ O_{6\times3} & O_{6\times9} & I_{6\times6} \end{matrix} \right]$$

$$\left\{ \begin{aligned} \begin{aligned} \begin{aligned} a_{17},a_{27}, a_{37}, a_{18},a_{28}, a_{38},a_{19},a_{29}, a_{39}, \\ a_{43},a_{44}, a_{45}, a_{46},a_{53}, a_{54},a_{55},a_{56}, a_{64}, \end{aligned} \\ a_{65},a_{66},a_{67},a_{57},a_{73},a_{83},a_{93},a_{103}, \end{aligned} \\ {a_{113},a}_{123},a_{91},a_{111},a_{33} \end{aligned} \right.=0$$

$$\left\{ \begin{aligned} a_{47},a_{52} \\ a_{63} \end{aligned}=1 \right.$$

$$a_{11}=s_{\phi}s_{\psi}v+c_{\phi}s_{\psi}w+c_{\phi}c_{\theta}v-c_{\psi}s_{\phi}s_{\theta}w$$

$$a_{21}=-c_{\psi}^{2}w+s_{\phi}^{2}w-s_{\phi}c_{\psi}v+c_{\phi}s_{\psi}s_{\theta}v-s_{\phi}s_{\psi}s_{\theta}w$$

$$a_{31}=c_{\theta}c_{\phi}v-c_{\theta}s_{\theta}w$$

$$a_{41}=t_{\theta}c_{\phi}q-t_{\theta}s_{\phi}r$$

$$a_{51}=-c_{\phi}r-s_{\phi}q$$

$$a_{61}=\frac{c_{\phi}}{c_{\theta}}q-\frac{s_{\phi}}{c_{\theta}}r$$

$$a_{71}=0$$

$$a_{81}=-50c_{\phi}c_{\theta}$$

$$a_{101}=-96c_{\phi}c_{\theta}$$

$$a_{121}=1.6c_{\phi}c_{\theta}$$

$$a_{12}=-c_{\phi}s_{\theta}u+c_{\phi}c_{\theta}c_{\phi}w+c_{\psi}c_{\theta}s_{\phi}v$$

$$a_{22}=-s_{\phi}s_{\theta}u+c_{\theta}s_{\psi}c_{\phi}w+c_{\theta}s_{\phi}v$$

$$a_{32}=-c_{\theta}u-s_{\theta}c_{\phi}w-s_{\theta}s_{\phi}v$$

$$a_{42}=\left( t_{\theta+1}c_{\phi} \right)r+\left( t_{\theta}^{2}+1 \right)s_{\phi}$$

$$a_{62}=r\left( \frac{s_{\theta}}{c_{\theta}^{2}}c_{\phi}+q\frac{s_{\theta}}{c_{\theta}^{2}}s_{\phi} \right)$$

$$a_{72}=1.1c_{\theta}$$

$$a_{82}=50 s_{\phi}s_{\theta}$$

$$a_{92}=-0.21 c_{\theta}$$

$$a_{102}=96 s_{\phi}s_{\theta}$$

$$a_{112}=-1.176c_{\theta}$$

$$a_{122}=-1.6s_{\phi}s_{\theta}$$

$$a_{13}=-s_{\psi}c_{\theta}u-c_{\phi}c_{\psi}v+c_{\psi}s_{\phi}v+c_{\psi}s_{\phi}w-s_{\phi}s_{\psi}s_{\theta}v-c_{\phi}s_{\psi}s_{\theta}w$$

$$a_{23}=c_{\psi}c_{\theta}u-c_{\phi}s_{\psi}v+c_{\phi}c_{\psi}s_{\theta}w+c_{\psi}s_{\theta}s_{\phi}$$

$$a_{14}=c_{\psi}c_{\theta}$$

$$a_{24}=c_{\theta}s_{\psi}$$

$$a_{34}=-s_{\theta}$$

$$a_{51}=-c_{\phi}s_{\psi}+c_{\psi}s_{\theta}$$

$$a_{52}=c_{\phi}c_{\psi}+s_{\phi}s_{\psi}s_{\theta}$$

$$a_{53}=c_{\theta}s_{\phi}$$

$$a_{61}=s_{\phi}s_{\psi}+c_{\phi}c_{\psi}s_{\theta}$$

$$a_{62}=-c_{\phi}s_{\phi}+c_{\phi}s_{\psi}s_{\theta}$$

$$a_{63}=c_{\phi}c_{\theta}$$

$$a_{77}=-0.032p-2r$$

$$a_{87}=-0.2q-0.9w$$

$$a_{97}=p-0.062r-v$$

$$a_{107}=-0.03q$$

$$a_{117}=-0.036p+1.34r$$

$$a_{127}=-0.77q$$

$$a_{48}=s_{\phi}t_{\theta}$$

$$a_{68}=c_{\phi}$$

$$a_{68}=\frac{s_{\phi}}{c_{\theta}}$$

$$a_{78}=0.632q+0.013u-1.8w$$

$$a_{88}=-0.0208p-12.5r$$

$$a_{98}=1.035q+0.57u+0.013w$$

$$a_{108}=-0.32p-23r$$

$$a_{118}=-0.026q-0.014u+0.074w$$

$$a_{128}=-0.77p+0.32r$$

$$a_{49}=c_{\phi}t_{\theta}$$

$$a_{59}=-s_{\phi}$$

$$a_{69}=\frac{c_{\phi}}{t_{\theta}}$$

$$a_{79}=-2.1p-0.66r+1.7v$$

$$a_{89}=-12.5q+1.25u$$

$$a_{99}=0.0617p-0.0109r-0.013v$$

$$a_{109}=-23q+3.5u$$

$$a_{119}=1.34p-0.062r-0.074v$$

$$a_{129}=0.323q-0.058u$$

$$a_{74}=a1*(Tu1)+a2*(Tu3)+a3*(Tu5)$$

$$a_{84}=b1*(Tu2)+b2*(Tu4)+b3*(Tu6)$$

$$a_{94}=a4*(Tu1)+a5*(Tu3)+a6*(Tu5)$$

$$a_{104}=b4*(Tu2)+b5*(Tu4)+b6*(Tu6)$$

$$a_{114}=a7*(Tu1)+a8*(Tu3)+a9*(Tu5)$$

$$a_{124}=b7*(Tu2)+b8*(Tu4)+b9*(Tu6)$$

$$a_{75}=a1*(Tv1)+a2*(Tv3)+a3*(Tv5)$$

$$a_{85}=b1*(Tv2)+b2*(Tw4)+b3*(Tv6)$$

$$a_{95}=a4*(Tv1)+a5*(Tv3)+a6*(Tv5)$$

$$a_{105}=b4*(Tv2)+b5*(Tv4)+b6*(Tv6)$$

$$a_{115}=a7*(Tv1)+a8*(Tv3)+a9*(Tv5)$$

$$a_{125}=b7*(Tv2)+b8*(Tv4)+b9*(Tv6)$$

$$a_{76}=a1*(Tw1)+a2*(Tw3)+a3*(Tw5)$$

$$a_{86}=b1*(Tw2)+b2*(Tw4)+b3*(Tw6)$$

$$a_{96}=a4*(Tw1)+a5*(Tw3)+a6*(Tw5)$$

$$a_{106}=b4*(Tw2)+b5*(Tw4)+b6*(Tw6)$$

$$a_{116}=a7*(Tw1)+a8*(Tw3)+a9*(Tw5)$$

$$a_{126}=b7*(Tu2)+b8*(Tu4)+b9*(Tu6)$$

$$Tu1=0$$

$$Tu2=-26.11r$$

$$Tu3=26.11q$$

$$Tu4=23.35r$$

$$Tu5=-79.44q$$

$$Tu6=-79.44r$$

$$Tv1=45r$$

$$Tv2=0$$

$$Tv3=-45p$$

$$Tv4=0$$

$$Tv5=7.9p+23.35r$$

$$Tv6=0$$

$$Tw1=-45q$$

$$Tw2=45p$$

$$Tw3=0$$

$$Tw4=23.35p$$

$$Tw5=-23.35q$$

$$Tw6=8p$$

$M^{-1}=\left[ \begin{matrix} a_{1} & 0 & a_{2} & 0 & a_{3} & 0 \\ 0 & b_{1} & 0 & b_{2} & 0 & b_{3} \\ a_{4} & 0 & a_{5} & 0 & a_{6} & 0 \\ 0 & b_{4} & 0 & b_{5} & 0 & b_{6} \\ a_{7} & 0 & a_{8} & 0 & a_{9} & 0 \\ 0 & b_{7} & 0 & b_{8} & 0 & b_{9} \end{matrix} \right]$
