# Supplementary material for: Unscented Kalman filter for airship model uncertainties and wind disturbance estimation
Source: PLoS One. 2021 Nov 5;16(11):e0257849. doi: 10.1371/journal.pone.0257849 (PMC8570505; doi:10.1371/journal.pone.0257849)
Supplement: S1 Nomenclature — (DOCX) [file pone.0257849.s003.docx]

Nomenclature and Acronyms

Acronyms

| BSC | Back-Stepping Controller |
| --- | --- |
| CG | Center of Gravity |
| CV | Center of Volume |
| CPU | Central Processing Unit |
| DOF | Degree Of Freedom |
| EKF | Extended Kalman Filter |
| GPS | Global Positioning System |
| IMU | Inertial Measurement Unit |
| NMPC | Nonlinear Model Predictive Controller |
| NED | North East Down |
| ROVs | Remotely Operated Underwater Vehicles |
| SMC | Sliding Mode Controller |
| UETT | University of Engineering and Technology, Taxila |
| UAV | Unmanned Aerial Vehicle |
| UT | Unscented Transformation |
| UKF | Unscented Kalman Filter |

Constants and Variables

| $\boldsymbol{P, \Phi}\boldsymbol{, \nu,}\boldsymbol{\Omega}$ | Airship position, attitudes, linear, and angular velocities, respectively |  |
| --- | --- | --- |
| $\boldsymbol{x, y,z}$ | Airship position coordinates with respect to x, y, and z axes | m |
| $\boldsymbol{\phi, \theta, \psi}$ | Roll, pitch, and yaw angles | rad |
| $\boldsymbol{u, v, w}$ | Forward, sway, and vertical velocity components | ms^-1^ |
| $\boldsymbol{p,q,r}$ | Roll, pitch, and yaw rates | rads^-1^ |
| $\bar{\boldsymbol{\xi}}$ | Vector of airship position and attitudes |  |
| ${\bar{\boldsymbol{V}}}_{\boldsymbol{b}}$ | Vector of airship body axes linear and angular velocities |  |
| $\boldsymbol{R}\left( \boldsymbol{\Phi} \right)$ | Rotation matrix that transforms linear and angular velocities from body axes to the inertial frame position and attitudes derivatives |  |
| ${\bar{\boldsymbol{F}}}_{\boldsymbol{D}}\boldsymbol{,}{\bar{\boldsymbol{F}}}_{\boldsymbol{AS}}\boldsymbol{,}{\bar{\boldsymbol{F}}}_{\boldsymbol{AD}}$ | Dynamic, aerostatic, and aerodynamic force vectors with uncertain modeling equations | N |
| $\boldsymbol{F}_{\boldsymbol{D}}\boldsymbol{,}\boldsymbol{F}_{\boldsymbol{AS}}\boldsymbol{,}\boldsymbol{F}_{\boldsymbol{AD}}$ | Dynamic, aerostatic, and aerodynamic force vectors with nominal parameters | N |
| $\boldsymbol{F}_{\boldsymbol{W}}$ | Vector of wind forces | N |
| $\boldsymbol{U}$ | Vector of control inputs |  |
| $\bar{\boldsymbol{M}}$ | Mass matrix with uncertain parameters | kg |
| $\boldsymbol{M}$ | Mass matrix with nominal parameters | kg |
| $\boldsymbol{\Delta}\boldsymbol{F}_{\boldsymbol{Mu}}$ | Uncertainty vector |  |
| $\boldsymbol{\Delta F}_{\boldsymbol{u}}\boldsymbol{,\Delta}\boldsymbol{F}_{\boldsymbol{v}}\boldsymbol{, \Delta}\boldsymbol{F}_{\boldsymbol{w}}$ | The uncertainty in forwarding, sway, and vertical acceleration, respectively | ms^-2^ |
| $\boldsymbol{\Delta F}_{\boldsymbol{p}}\boldsymbol{,}\boldsymbol{\Delta F}_{\boldsymbol{q}}\boldsymbol{,}\boldsymbol{\Delta F}_{\boldsymbol{r}}$ | The uncertainty in angular roll, pitch, and yaw acceleration, respectively | rads^-2^ |
| $\boldsymbol{Q,R,}\boldsymbol{P}_{\boldsymbol{k}}$ | Process, measurement, and state error covariance |  |
| $\boldsymbol{V}_{\boldsymbol{t}}$ | Airship velocity | ms^-1^ |
| $\boldsymbol{m}$ | Airship total mass | kg |
| $\boldsymbol{O}_{\boldsymbol{E}}\boldsymbol{X}_{\boldsymbol{E}}\boldsymbol{Y}_{\boldsymbol{E}}\boldsymbol{Z}_{\boldsymbol{E}}$ | Inertial or NED axes system |  |
| $\boldsymbol{O}_{\boldsymbol{b}}\boldsymbol{X}_{\boldsymbol{b}}\boldsymbol{Y}_{\boldsymbol{b}}\boldsymbol{Z}_{\boldsymbol{b}}$ | Body axes system |  |
| $\boldsymbol{m}_{\boldsymbol{x}}\boldsymbol{,}\boldsymbol{m}_{\boldsymbol{y}}\boldsymbol{,}\boldsymbol{m}_{\boldsymbol{z}}$ | Airship mass components along x, y, and z axes in the body frame of reference | kg |
| $\boldsymbol{\alpha}$ | Angle of attack | rad |
| $\boldsymbol{\beta}$ | Sideslip angle | rad |
| $\boldsymbol{\rho}$ | Air density | kg/m^3^ |
| S | Total surface area of airship | m^2^ |
| W | Weight of airship | N |
| $\boldsymbol{B}_{\boldsymbol{f}}$ | Buoyancy of airship | N |
| d | Airship diameter | m |
| l | Airship length | m |
| $\boldsymbol{J}_{\boldsymbol{x}}\boldsymbol{,}\boldsymbol{J}_{\boldsymbol{y}}\boldsymbol{,}\boldsymbol{J}_{\boldsymbol{z}}\boldsymbol{,}\boldsymbol{J}_{\boldsymbol{xz}}$ | Airship Inertia components along x, y and z axes in body frame of reference | kg |
| $\boldsymbol{a}_{\boldsymbol{x}}\boldsymbol{,}\boldsymbol{a}_{\boldsymbol{z}}$ | Coordinates of airship CG | m |
| $\boldsymbol{b}_{\boldsymbol{x}}\boldsymbol{,}\boldsymbol{b}_{\boldsymbol{z}}$ | Coordinates of airship center of buoyancy | m |
| $\boldsymbol{C}_{\boldsymbol{X}\boldsymbol{1}}\boldsymbol{,}\boldsymbol{C}_{\boldsymbol{X}\boldsymbol{2}}$ | Aerodynamic drag force coefficients |  |
| $\boldsymbol{C}_{\boldsymbol{Y}\boldsymbol{1}}\boldsymbol{,}\boldsymbol{C}_{\boldsymbol{Y}\boldsymbol{2}}\boldsymbol{,}\boldsymbol{C}_{\boldsymbol{Y}\boldsymbol{3}}\boldsymbol{,}\boldsymbol{C}_{\boldsymbol{Y}\boldsymbol{4}}$ | Aerodynamic side force coefficients |  |
| $\boldsymbol{C}_{\boldsymbol{Z}\boldsymbol{1}}\boldsymbol{,}\boldsymbol{C}_{\boldsymbol{Z}\boldsymbol{2}}\boldsymbol{,}\boldsymbol{C}_{\boldsymbol{Z}\boldsymbol{3}}\boldsymbol{,}\boldsymbol{C}_{\boldsymbol{Z}\boldsymbol{4}}$ | Aerodynamic lift force coefficients |  |
| $\boldsymbol{C}_{\boldsymbol{L}\boldsymbol{1}}\boldsymbol{,}\boldsymbol{C}_{\boldsymbol{L}\boldsymbol{2}}$ | Aerodynamic roll moment coefficients |  |
| $\boldsymbol{C}_{\boldsymbol{M}\boldsymbol{1}}\boldsymbol{,}\boldsymbol{C}_{\boldsymbol{M}\boldsymbol{2}}\boldsymbol{,}\boldsymbol{C}_{\boldsymbol{M}\boldsymbol{3}}\boldsymbol{,}\boldsymbol{C}_{\boldsymbol{M}\boldsymbol{4}}$ | Aerodynamic pitch moment coefficients |  |
| $\boldsymbol{C}_{\boldsymbol{N}\boldsymbol{1}}\boldsymbol{,}\boldsymbol{C}_{\boldsymbol{N}\boldsymbol{2}}\boldsymbol{,}\boldsymbol{C}_{\boldsymbol{N}\boldsymbol{3}} \boldsymbol{C}_{\boldsymbol{N}\boldsymbol{4}}$ | Aerodynamic yaw moment coefficients |  |
| $\boldsymbol{V}_{\boldsymbol{w}}$ | Vector of wind velocities in an inertial frame | ms^-1^ |
| $\boldsymbol{L}_{\boldsymbol{u}}\boldsymbol{,}\boldsymbol{L}_{\boldsymbol{v}}\boldsymbol{,}\boldsymbol{L}_{\boldsymbol{w}}$ | Turbulence scale lengths |  |
| $\boldsymbol{\sigma}_{\boldsymbol{u}}\boldsymbol{,}\boldsymbol{\sigma}_{\boldsymbol{v}}\boldsymbol{,}\boldsymbol{\sigma}_{\boldsymbol{w}}$ | Intensities of turbulence |  |
| $\boldsymbol{u}_{\boldsymbol{g}}\boldsymbol{,}\boldsymbol{v}_{\boldsymbol{g}}\boldsymbol{,}\boldsymbol{w}_{\boldsymbol{g}}$ | Atmospheric gust components | ms^-1^ |
| $\boldsymbol{V}_{\boldsymbol{g}}$ | Gust velocity vector | ms^-1^ |
| $\boldsymbol{V}_{\boldsymbol{a}}$ | Airflow velocity vector | ms^-1^ |

Statistical Terms

| $\boldsymbol{\sigma}$ | Standard deviation of error |
| --- | --- |
| $\boldsymbol{e}_{\boldsymbol{i}}$ | Error in estimate |
| $\boldsymbol{D}_{\boldsymbol{FB}}$ | Distance of error exceeding the uncertainty bound |
| $\boldsymbol{P}_{\boldsymbol{e}}$ | Percentage of error exceeding the uncertainty bound |
| $\boldsymbol{M}_{\boldsymbol{e}}$ | Mean estimation error |
